# Surface-Exposed Hydroxyapatite Microparticles in Electrospun PLLA Scaffolds: Mechanical Reinforcement and Osteogenic Response

**DOI:** 10.3390/polym18162001

**Published:** 2026-08-17

**Authors:** Arsalan D. Badaraev, Mikhail A. Buldakov, Vladislav R. Bukal, Evgeny L. Choinzonov, Sven Rutkowski, Xiaojun Han, Sergei I. Tverdokhlebov

**Affiliations:** 1 State Key Laboratory of Urban-Rural Water Resource and Environment, Heilongjiang Provincial Joint Laboratory of Molecular Science (International Cooperation), School of Chemistry and Chemical Engineering, Harbin Institute of Technology, Harbin 150001, China; adb6@tpu.ru; 2 Weinberg Research Center, School of Nuclear Science & Engineering, National Research Tomsk Polytechnic University, 30, Lenin Avenue, 634050 Tomsk, Russia; vrb2@tpu.ru (V.R.B.); sven-rut@gmx.de (S.R.); 3 Cancer Research Institute, Tomsk National Research Medical Center, Russian Academy of Sciences, 5 Per. Kooperativny, 634050 Tomsk, Russia; buldakov@oncology.tomsk.ru (M.A.B.); choynzonov@tnimc.ru (E.L.C.)

**Keywords:** electrospinning, hydroxyapatite, exposed microparticles, poly-L-lactide, osteogenic tissue regeneration

## Abstract

The addition of hydroxyapatite (HAP) to electrospun poly-L-lactide (PLLA) scaffolds promotes cell adhesion and differentiation but generally leads to a significant deterioration in mechanical properties due to particle agglomeration. Moreover, the encapsulation of HAP particles within a polymer layer makes them inaccessible to body fluids and cells, thereby limiting the bioactivity of the resulting composite scaffold. In this work, HAP microparticles with median size of 26.3 µm were used to obtain exposed HAP particles on the surface of electrospun PLLA fibers. SEM images and EDX maps revealed that individual particles, particularly the larger ones, were exposed from the polymer scaffold surface. The addition of HAP particles significantly altered the scaffold morphology and structure, increasing the fiber diameter and surface roughness by 2.8–4.1-fold, promoting the formation of fused fiber junctions, and inducing the appearance of semicrystalline PLLA domains. These structural changes significantly improved the mechanical properties of the scaffolds. Specifically, the tensile strength and Young’s modulus of the prepared scaffolds are increased by 2.3–3.8-fold following HAP incorporation. Compared with neat PLLA scaffolds, HAP-containing scaffolds exhibited 1.2–1.4-fold higher osteocalcin and osteopontin expression by human adipose-derived mesenchymal stromal cells (hADSCs). Compared to tissue culture plastic, the expressions of osteocalcin and osteopontin on the composite scaffolds were 7.1–7.9-fold and 2.8–3.0-fold higher, respectively.

## 1. Introduction

Electrospun scaffolds are widely used in tissue engineering because they provide flexible, mechanically resistant, and highly porous structures that mimic the architecture of the extracellular matrix and promote cell adhesion and migration [1,2,3,4,5]. Among polymer-ceramic systems developed for bone tissue engineering, the electrospun poly(L-lactide) (PLLA)/hydroxyapatite (HAP) scaffolds rank as the most widely studied [6]. PLLA is the L-stereoisomer of polylactide (PLA) and possesses bioresorbable and biocompatible properties [6] with intrinsic piezoelectric properties, that positively affect cell proliferation, differentiation [7] and adhesion [8]. Hydroxyapatite is the main component of bone tissue [9] and is formed in the living body by osteoblasts [10]. Owing to its close compositional similarity to native bone mineral, HAP promotes the osteogenic differentiation of stem cells [11]. The presence of HAP enhances the biological activity and the osteogenesis of stem cells, which supports the formation of new bone tissue [12,13,14]. Accordingly, PLLA/HAP composite scaffolds have repeatedly demonstrated their effectiveness in bone tissue regeneration [15,16,17].

During the production of PLLA/HAP composite scaffolds using electrospinning, hydroxyapatite particles are frequently encapsulated within the polymer phase rather than exposed on their surfaces [18,19]. This behavior may be attributed to interfacial interactions between polylactide and the surface of hydroxyapatite particles. According to molecular dynamics simulations, the oxygen-containing groups of PLLA form coordination bonds with calcium ions on the HAP surface, resulting in increased Ca-O coordination numbers and a higher interfacial density at the PLLA/HAP interface [20]. These interactions promote better adhesion and attachment of polymer on HAP particles, facilitate stress transfer between the filler and the polymer phase, and contribute to the mechanical reinforcement of the final polymer-ceramic composite.

However, the availability of HAP particles and their exposure to the scaffold surface is essential for achieving positive biological performance. When HAP particles are completely encapsulated by the polymer, direct interactions between the mineral phase and cells or biological fluids are greatly reduced [21]. As a result, protein adsorption, calcium and phosphorus ion exchange, formation of the apatite layer, and osteogenic differentiation are significantly hampered, limiting the bioactivity of the composite scaffold. Another important factor is the deterioration of the mechanical properties of polymer-HAP composites with increasing of HAP content [22,23]. This effect is especially critical for fibrous materials produced by electrospinning, in which even a modest increase in the concentration of HAP can significantly reduce tensile strength. For example, Lopresti et al. reported that electrospun PLA/HAP scaffolds containing 10 wt.% HAP exhibited a tensile strength approximately three times lower than that of neat PLA scaffolds [24]. This reduction in mechanical properties limits the degree of effective filling of the polymer matrix with hydroxyapatite. As a result, achieving an electrospun scaffold with HAP loading more than 10–20 wt.% while maintaining or improving its mechanical properties is a challenging task. This is significant, since poor mechanical properties of HAP-containing electrospun PLA scaffolds may limit cell proliferation and differentiation [25,26]. For example, PLA scaffolds containing 6 wt.% and 10 wt.% HAP exhibit superior mechanical and biological properties, whereas increasing the HAP content to 20 wt.% reduced the number of proliferated cells and alkaline phosphatase activity by approximately twofold, which was attributed to the poor mechanical properties of prepared scaffolds [25]. This effect is strongly pronounced for HAP nanoparticles with high specific surface area. Their strong agglomeration tendency promotes the formation of stress concentration points and a deterioration in mechanical properties, making HAP-containing polymer material brittle [25,27]. Furthermore, the high specific surface area of HAP nanoparticles enhances their interactions with the polymer solution or melt, increasing the probability of complete polymer encapsulation of the mineral particles and formation of a polymer coating on the particle surface [18], thereby limiting their accessibility and bioavailability to cells. The use of HAP microparticles may provide a simple strategy for increasing the loading of mineral phase in polymer scaffold without a substantial loss of mechanical properties. Compared with HAP nanoparticles, microparticles have smaller specific surface area and, correspondingly, a smaller polymer-particle interfacial contact area per unit mass of filler. This reduced interfacial area may decrease the encapsulation efficiency of HAP by polymer, which potentially will preserve HAP accessibility to cells. As a result, the use of HAP microparticles potentially allows for an increase in the effective loading of the bioactive mineral phase without completely shielding its surface with the polymer matrix. Moreover, for a given mineral phase loading, the required number of HAP microparticles is substantially lower than that of HAP nanoparticles. Assuming particles of similar shape and density, the number of particles is inversely proportional to the cube of their diameter (N ∝ 1/d^3^). Therefore, a tenfold increase in particle diameter reduces the required number of particles by approximately three orders of magnitude (1000-fold), which substantially decreases the probability of particle–particle contacts and, consequently, the likelihood of agglomeration. Reduced agglomeration is expected to minimize stress concentration points within polymer, thereby helping to preserve the mechanical integrity of the scaffold without significant embrittlement. A similar concept was used by Metwally et al. [28], who incorporated mineral microparticles with a mean diameter of 1.2  ±  0.1 μm into electrospun polycaprolactone scaffolds. The resulting composite scaffolds demonstrated substantially higher tensile strength and Young’s modulus than the neat polymer scaffolds. In addition, some mineral microparticles were protruded from the surface of the polymer fibers, indicating not full polymer encapsulation.

Therefore, the aim of this study was to fabricate electrospun composite scaffolds containing HAP microparticles using a one-step preparation process to achieve high mineral phase loading, improved mechanical strength, and a significant fraction of surface-exposed HAP particles. It is assumed that the lower specific surface area of HAP microparticles, together with the substantially smaller number of particles required to achieve a given mineral phase loading, would reduce particle–particle interactions and agglomeration while simultaneously decreasing the probability of complete HAP encapsulation by polymer. Consequently, the loading degree of the mineral phase without significant embrittlement increases, and partially exposed bioactive regions of HAP will be present on the fiber surface.

## 2. Materials and Methods

### 2.1. Materials and Spinning Solution Preparation

Firstly, poly-L-lactide (PLLA) granules (intrinsic viscosity—3.8 dL/g, PURASORB^®^ PL-38, Purac, Amsterdam, The Netherlands) were dissolved in trichloromethane (CHCl_3_, purity 98.5%, Ekos-1, Moscow, Russia) in an amount of 3 wt.% of the CHCl_3_ weight. After complete dissolution of PLLA, acetone (C_3_H_6_O, purity 99.8%, Ekos-1, Moscow, Russia) was added to CHCl_3_ at 30 *v*/*v*% of the obtained PLLA solution. Subsequently, hydroxyapatite (HAP) microparticles (Ca_10_(PO_4_)_6_(OH)_2_, CaHAP-DP, BITECA LLC, Odintsovo, Russia) were added to the PLLA solution in an amount of 20 wt.% and 30 wt.% of the PLLA weight, which corresponds to ~16.7 wt.% and ~23.1 wt.% of the total mass of solids (PLLA and HAP), respectively. The median size of utilized HAP particles was 26.3 µm (Q1: 21.4 µm, Q3: 31.0 µm). The obtained solutions were stirred at room temperature and at 800 rpm for 10 min. Prior to the electrospinning process, to ensure uniform distribution of HAP particles in the solution, vessels containing PLLA/HAP spinning solutions were placed in an ultrasonic bath (UZV-4,0/1 “Sapphire” TTC RMD, Moscow, Russia) for 30 min at room temperature.

### 2.2. Preparation of Composite PLLA/HAP Scaffolds

For the electrospinning process, the PLLA/HAP solutions were loaded in an electrospinning device (NANON-01 A, MECC CO, Fukuoka, Japan). The following technological modes were used during electrospinning: solution flow rate—6 mL/h; applied voltage—+20 kV, distance between needle and collector—180 mm, needle size—gauge 22; spinneret speed—10 mm/min, rotational speed of collector—50 rpm. During the electrospinning process, a cylindrical collector with a diameter of 100 mm and width of 210 mm was used, with an average temperature and humidity of (27.8 ± 1.7) °C and (19 ± 2)%, respectively. The fabricated scaffolds had an average thickness of 263 ± 30 µm.

The following designations for the scaffold samples are used below: “PLLA”—electrospun PLLA scaffolds without HAP; “16.7%”—electrospun PLLA/HAP scaffolds containing 20 wt.% HAP relatives to the PLLA mass; “23.1%”—electrospun PLLA/HAP scaffolds containing 30 wt.% HAP relatives to the PLLA mass.

### 2.3. Characterization Methods

#### 2.3.1. Electrical Conductivity and Viscosity of Spinning Solutions

The conductivity values of PLLA and PLLA/HAP spinning solutions were estimated via an inoLab Cond 7319 conductometer (WTW, Weilheim, Germany) equipped with a TetraCon 325 universal conductivity measuring cell (WTW, Weilheim, Germany). The viscosity values of the spinning solutions were measured using a viscometer (SV-10, A&D, Tokyo, Japan). The measurements were conducted at room conditions.

#### 2.3.2. Bright-Field Microscopy

A bright-field microscope (DM-1802 Motic, Xiamen, Fujian, China) with at ×4 and ×10 magnifications was used to study the morphology of the electrospun scaffold samples. Distribution histograms of HAP particles were estimated manually using ImageJ 1.51w software (National Institutes of Health, Washington, DC, USA) by measuring ~200 distinct particles at ×10 magnification for samples at 16.7% and 23.1%, and images of only hydroxyapatite particles in bright field.

#### 2.3.3. Scanning Electron Microscopy

Prior to investigation, a thin layer of gold was deposited on the surfaces of the composite PLLA/HAP scaffolds using a sputtering device (SmartCoater, Jeol, Akishima, Japan) to provide charge dissipation. The morphology of the PLLA/HAP scaffold surfaces was investigated using a scanning electron microscope (SEM, ESEM Quanta 200 3D, FEI Company, Hillsboro, OR, USA) at a magnification of ×200 and ×1000 and at an accelerating voltage of 20 kV under high vacuum mode. Distribution histograms of fiber diameter were estimated manually using ImageJ 1.51w software (National Institutes of Health, Washington, DC, USA) by measuring ~200 fiber diameter values for each sample. Finally, the histograms for pore area distribution were determined using ImageJ 1.51w according to the methodology described in ref. [29].

#### 2.3.4. Optical Profilometry

Interferometric surface relief, arithmetic mean roughness (*R_a_*) and average peak-to-valley roughness (*R_z_*) were estimated using an optical profilometer MNP-1 (Technological Design Institute of Scientific Instrument Engineering SB RAS, Novosibirsk, Russia). For the analysis, the following parameters of the optical profilometer were used: scanning range—200 µm, scanning step—0.5 µm, and minimum threshold detection—2.7.

#### 2.3.5. Porosity and Areal Density

The gravimetric method was used to determine the porosity (*P*) of the scaffolds. The porosity was calculated according to the following equation [30]:

(1) $$P=(1-\frac{\rho_{scaffold}}{\rho_{solid}})\cdot 100\%,$$

where $\rho_{scaffold}$ is the density of the prepared scaffold (g/cm^3^), and $\rho_{solid}$ is the theoretical density of the corresponding non-porous bulk material.

The theoretical densities used for the calculations were 1.27 g/cm^3^ for PLLA [31] and 3.16 g/cm^3^ for HAP [32]. For PLLA/HAP scaffolds, the theoretical density was calculated based on the corresponding weighted fractions of PLLA and HAP.

The areal density was calculated as the mass of the scaffold divided by its projected surface area.

#### 2.3.6. Elemental Analysis Measurements

The elemental composition of the fabricated PLLA/HAP scaffolds was measured by energy-dispersive X-ray spectrometer (EDX, Genesis4000, Oxford Instruments, Abingdon, UK) integrated into a scanning electron microscope (SEM, ESEM Quanta 200 3D, FEI Company, Hillsboro, OR, USA). Analysis was corrected using the ZAF-correction (Z—atomic number, A—absorption effect and F—fluorescence excitation effect).

Additionally, the elemental composition of the prepared samples was evaluated using an X-ray fluorescence spectrometer (XRF, XRF 1800, Shimadzu, Kyoto, Japan), which was set to the following parameters: scanning speed—8°/min, and scanning step—0.1°. The analysis was performed using four elemental channels: carbon, oxygen, calcium, and phosphorus.

Elemental mapping of scaffold surfaces was also performed using abovementioned EDX system integrated with a scanning electron microscope. SEM images and elemental maps were acquired at a magnification of ×200 and ×1000 using an accelerating voltage of 20 kV. The signals of the following elements were gathered: carbon (C), oxygen (O), calcium (Ca), and phosphorus (P).

The surface area occupied by HAP particles on the prepared PLLA/HAP scaffolds was calculated based on EDX elemental maps images of the calcium (Ca) signal acquired at ×1000 magnification. The area occupied by HAP particles was quantitatively calculated using ImageJ 1.51w with a built-in “Analyze Particles” function.

For the analysis of HAP particles distribution on the surface of PLLA/HAP scaffolds, EDX elemental maps of the Ca signal at ×200 magnifications were used. The following quantitative parameters characterizing the spatial and non-spatial distribution of HAP particles were calculated: the coefficient of variation (*CV*), Gini coefficient (*G*) and Global Moran’s I (*I*). The following formulas were used for calculations [33,34,35]:

(2) CV=σ/μ·100%,

where σ is the standard deviation of the Ca signal intensity, and μ is the mean Ca signal intensity.

(3) $$G=\frac{2\sum_{i=1}^{N}i\cdot x_{i}}{N\sum x_{i}}-\frac{N+1}{N},$$

where *N* is the total number of grid cells, and $x_{i}$ is Ca signal intensity in the *i*th cell.

(4) $$I=\frac{N}{S_{0}}\frac{\sum_{i=1}^{N}\sum_{j=1}^{N}w_{ij}(x_{i}-\bar{x})(x_{j}-\bar{x})}{\sum_{i=1}^{N}{\left(x_{i}-\bar{x}\right)}^{2}},$$

where *N* is the number of spatial units, *x_i_* and *x_j_*—are the Ca signal intensities in the *i*th and *j*th spatial units, respectively, $\bar{x}$—is the mean Ca signal intensity, *w_ij_*—elements of spatial weight matrix with zeros on the diagonal, and $S_{0}=\sum_{i=1}^{N}\sum_{j=1}^{N}w_{ij}$.

For the analysis with the abovementioned formulas, each EDX map was partitioned into regular grids consisting of 8 × 8, 16 × 16, 32 × 32, or 64 × 64 cells. The corresponding parameters were calculated for each grid size.

#### 2.3.7. X-Ray Diffraction (XRD)

The structure of the prepared composite PLLA/HAP scaffolds was analyzed using an X-ray diffractometer (XRD 6000, Shimadzu, Kyoto, Japan) under the following parameters: X-ray tube with monochromatic Cu kα radiation at a wavelength of 1.54 Å; an X-ray tube voltage of 40 kV; an X-ray tube current of 30 mA; a scanning range of (10–80)°; a scanning speed of 2°/min; and a scanning step of 0.02°. The area of the samples was 1 × 1 cm^2^.

The average crystallite size (*D*) of PLLA and HAP crystallites were estimated using the following formula [36]:

*D* = *Kλ*/*(β cosθ)*, (5)

where *K*—dimensionless crystallite shape factor (K = 0.9), *λ*—X-ray irradiation wavelength (λ(Cu Kα) = 1.5406 Å), *β*—full width at half maximum (FWHM) of crystalline peak in radians, and *θ*—Bragg angle.

The crystallinity degree of PLLA was estimated as the ratio of the crystalline PLLA peaks area and the total area of the background-corrected diffraction profile [37].

#### 2.3.8. Thermogravimetric Analysis (TGA)

A thermal analyzer (SDT-Q600, TA Instruments, New Castle, DE, USA) was used to conduct thermogravimetric analysis of the prepared composite PLLA/HAP scaffolds. Weight of the samples investigated ranged from 10 mg to 17 mg. Samples were introduced in a heat-resistant vessel that was placed in a thermal analyzer, then the temperature increased from room temperature to 800 °C at a heating rate of 20 °C/min under atmospheric air.

#### 2.3.9. Wettability Measurements

To determine the water contact angle (WCA) values, a drop shape analyzer (DSA 20, Krüss, Hamburg, Germany) was used. The WCAs were measured after 1 min of interaction of the droplets with the top and bottom surfaces of each prepared PLLA/HAP scaffold. Three water droplets, each with a volume of 2 μL, were deposited to the surface of each individual sample with an area of 3 × 1 cm^2^.

#### 2.3.10. Mechanical Properties

The mechanical properties of the prepared scaffolds were tested on a tensile testing machine (Instron 3343, Illinois Tool Works, Glenview, IL, USA) employing a static 50 N load cell (Instron 2519-102, Illinois Tool Works, Glenview, IL, USA). Each PLLA/HAP scaffold sample had dimensions of 3 × 1 cm^2^, and its length was cut to match the direction of rotation of the cylindrical collector of the electrospinning device. The distance between the traverse jaws was 1 × 1 cm^2^ and the speed of traverse was 20 mm/min. Each sample type was measured in quintuplicate (*n* = 5).

#### 2.3.11. Biological Activity Assays

To evaluate the biological properties, cell studies were carried out using human adipose-derived stromal/stem cells (hADSCs) obtained by the enzymatic method from lipoaspirate collected during liposuction procedures performed on patients of the Oncology Research Institute, Tomsk National Research Medical Center of Russian Academy of Science (Tomsk, Russia). The hADSCs isolation was provided in accordance with Bioethics protocol (Supporting Information (SI) Figure S1, permission no. 11 dated 23 October 2015). Cell dissociation was performed using collagenase (50 mg, PanEco, Moscow, Russia). The cells obtained were incubated at 37 °C in a 5% CO_2_ atmosphere in complete DMEM/F12 culture medium (Biolot, St. Petersburg, Russia) supplemented with 10% fetal bovine serum (HyClone Laboratories Inc., Logan, UT, USA) and antibiotics: penicillin—5000 U/mL and streptomycin—5000 µg/mL (both purchased from PanEco, Moscow, Russia).

20 µL of cell suspension containing 10^5^ mesenchymal stem cells was applied to the surface of each sample. Cells were counted with automatic cell counter Countess II FL (Thermo Fisher Scientific, Waltham, MA, USA).

Cell adhesion was evaluated by fluorescent staining of the cells directly on the tested samples using the nuclear dye DAPI (Invitrogen, Waltham, MA, USA). Visualization was performed using the EVOS M7000 imaging system (Thermo Fisher Scientific, Waltham, MA, USA) with a 10× objective. Cell counting was carried out in 10 different fields of view on the sample surface area of 1100 × 1100 µm^2^.

Cytotoxic effects were assessed using an annexin V/propidium iodide assay kit (Elabscience Biotechnology Co., Ltd., Wuhan, Hubei, China), which can detect cells displaying signs of apoptosis and necrosis. Annexin V-positive cells were stained green, indicating early apoptosis. Cells that reacted positively to propidium iodide turned red, indicating necrotic cell death. Cells that were positive for both dyes appeared orange, indicating late apoptosis.

Differentiation at the osteoblast formation stage was evaluated on day 14 by assessing the expression of osteocalcin (BGLAP) and osteopontin (SPP1) genes using real-time polymerase chain reaction (RT-PCR).

### 2.4. Statistics

Statistical data analysis was conducted using OriginPro^®^ 2024 software (Origin-Lab, Northampton, MA, USA). Statistical differences in the morphology (fiber diameter, pore area, and roughness), in the mechanical and biological properties of PLLA and PLLA/HAP scaffolds were evaluated using one-way ANOVA and Kruskal–Wallis tests. The normality of the distribution was assessed using the Shapiro–Wilk test. Differences were considered statistically significant at *p* < 0.05.

### 2.5. Experimental Scheme

**Figure 1:**
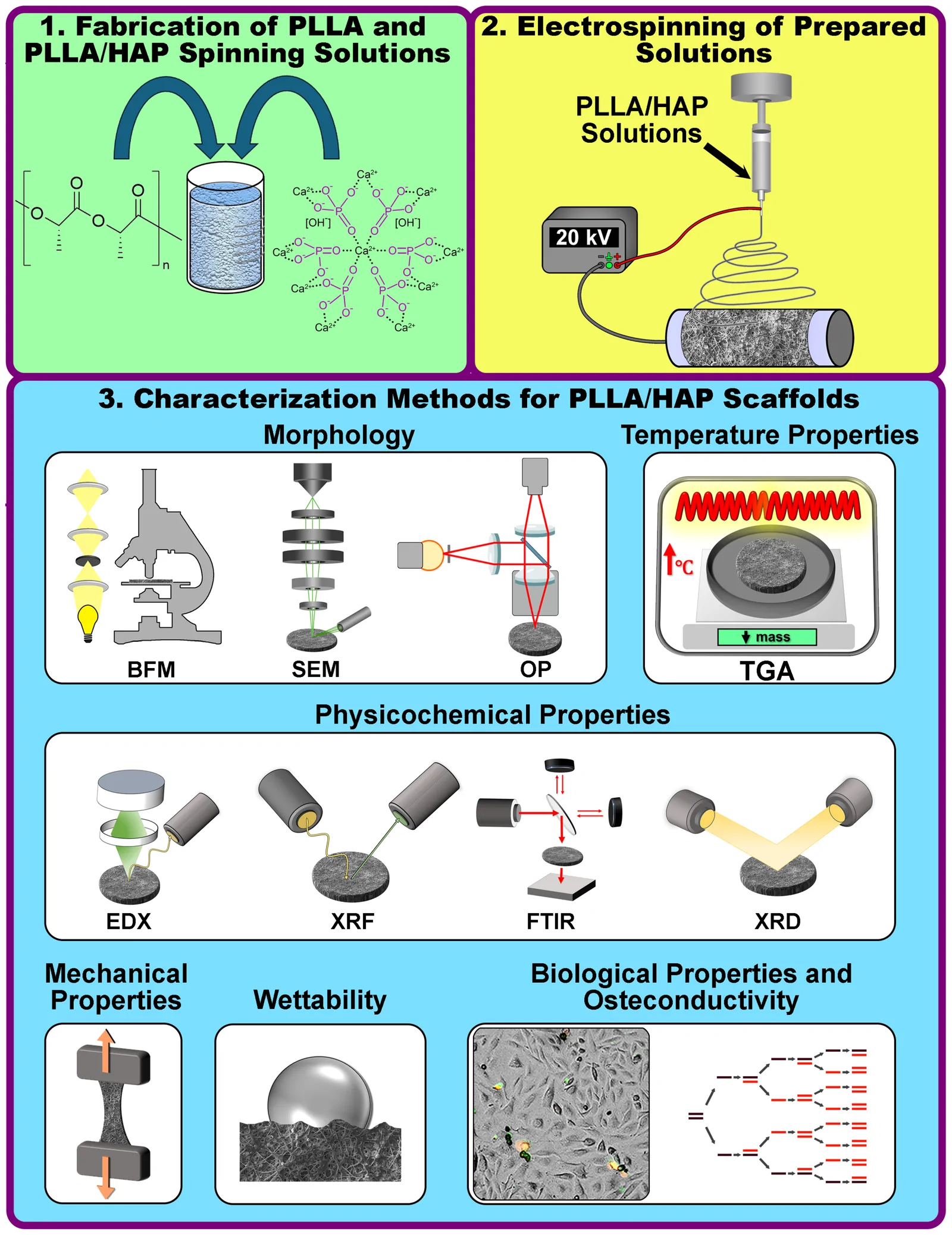
Experimental scheme for the fabrication of poly-L-lactide (PLLA)/hydroxyapatite (HAP) scaffolds and the further applied investigation methods in this study. In the first step, PLLA solutions and PLLA/HAP spinning dispersions were prepared in organic solvents (upper left corner). For the second step, PLLA solutions and PLLA/HAP dispersions were electrospun using an electrospinning device (upper right corner). Finally, the morphological, physico-chemical, biological and osteogenic properties of the prepared scaffolds were investigated (lower section). The abbreviations refer to: BFM—bright-field microscopy, SEM—scanning electron microscopy, OP—optical profilometry, TGA—thermogravimetric analysis, EDX—energy dispersive X-ray spectroscopy, XRF—X-ray fluorescence, FTIR—Fourier transform infrared spectroscopy, and XRD—X-ray diffraction.

An illustrative scheme that demonstrates the individual steps involved in the production of electrospun PLLA and PLLA/HAP scaffolds, as well as the applied investigation methods are presented in Figure 1.

## 3. Results and Discussion

### 3.1. Electroconductivity and Viscosity of Spinning Solutions

The electrical conductivity of the PLLA solution is equal to 0.2 µS/cm, while the conductivity of PLLA/HAP dispersions showed lower values between 0.0 and 0.1 µS/cm (SI Figure S2a). The decrease in electroconductivity after the addition of HAP particles is likely attributed to the adsorption of ionic residuals via surface of HAP particles, as hydroxyapatite is known to adsorb anions of organic nature [38,39]. In addition, acetone is component in the solution that gives a major contribution to the solution conductivity, while trichloromethane exhibits markedly lower conductivity [40]. Calcium ions (Ca^2+^) from HAP may interact with carbonyl oxygen of acetone [41], and surficial Ca^2+^ sites in HAP may act as Lewis-acidic centers [42]. Possible interaction of large HAP particles with acetone may restrict solvent mobility in spinning solution, further lowering electrical conductivity.

The viscosity of the pure PLLA solution is equal to 220 mPa·s, whereas after HAP addition, the solution viscosity slightly changes to 215 mPa·s and increases to 251 mPa·s for the “16.7%” and “23.1%” samples, respectively (SI Figure S2b). Similar findings were reported in [28,43], where it was demonstrated that HAP addition increases the spinning solution viscosity. The increase in viscosity at higher HAP content can be attributed to increased interfacial interactions between HAP and PLLA chains. Theoretical studies demonstrated that coordination interactions may occur between Ca^2+^ ions on the HAP surface and carbonyl (C=O) groups of PLLA with forming of Ca-O coordination bonds [20], which can possibly lead to restricted chain mobility and increased solution viscosity. Furthermore, HAP and PLLA can interact with each other through hydrogen bonding between ester carbonyl groups and surface of P-OH groups of HAP [44].

### 3.2. Physicochemical Properties of the PLLA and PLLA/HAP Scaffold Samples

**Figure 2:**
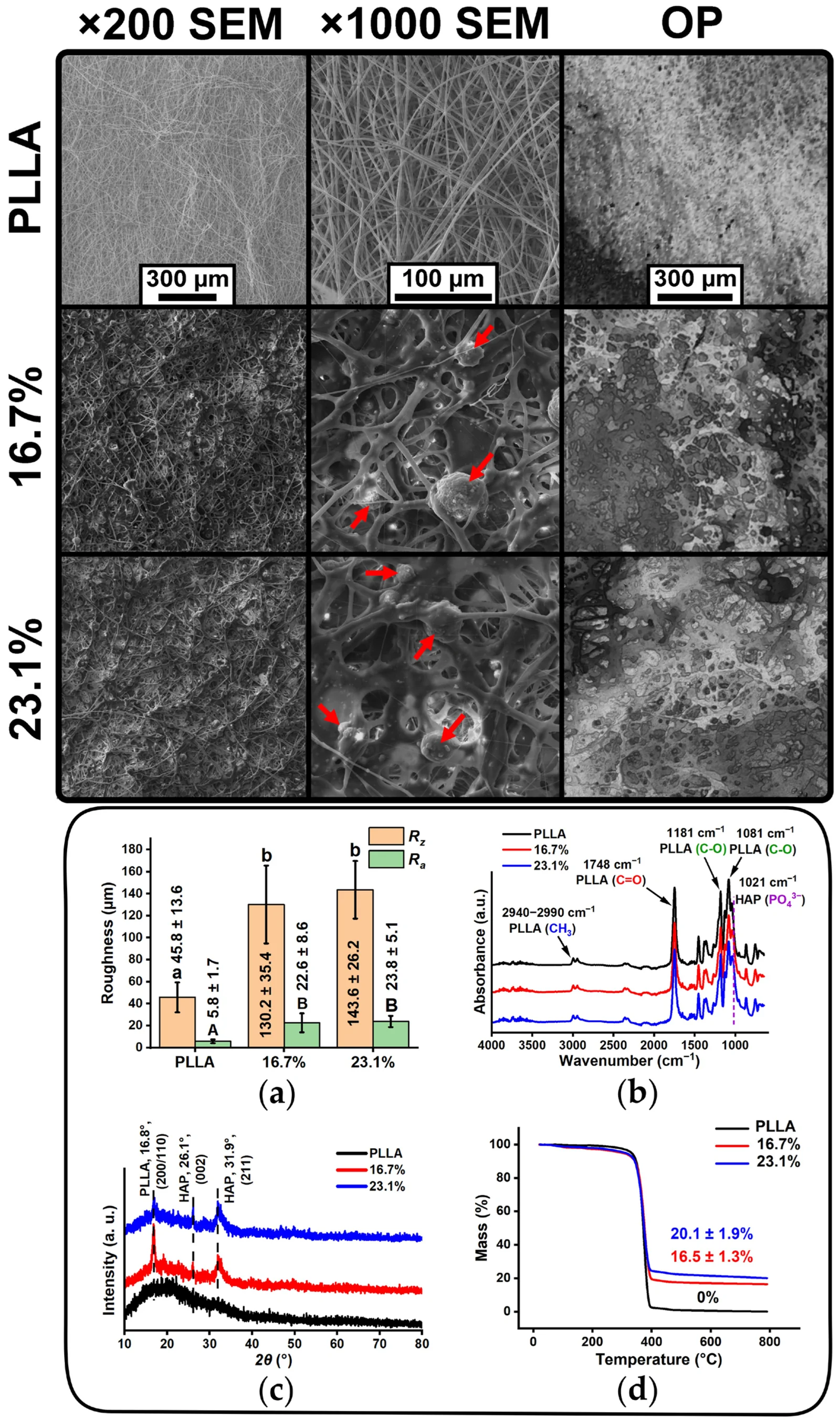
SEM and OP images (upper side of the picture), as well as the morphological, structural, thermal properties and qualitative chemical composition of PLLA and PLLA/HAP scaffolds (lower part of the picture): (**a**) Roughness values determined using an OP, (**b**) FTIR spectra, (**c**) XRD spectra, (**d**) TG curves reflecting the loss of mass with increasing temperature. Note: The red arrows on upper side of the image point to HAP particles. Lowercase and uppercase letters in figure (**a**) denote results of statistical comparisons, with different letters (e.g., a, b or A, B) indicating statistically significant differences (*p* < 0.05).

The scanning electron microscopy (SEM) images at ×200 and ×1000 magnification, optical profilometry (OP) images, roughness values, calculated based on OP images, Fourier infrared spectra (FTIR), X-ray diffraction (XRD) spectra and thermogravimetric (TG) curves of PLLA and PLLA/HAP scaffolds are presented in Figure 2.

Electrospun PLLA scaffolds exhibited nonwoven morphology (Figure 2, SEM images) with a median fiber diameter of 2.6 µm (Q1: 2.1 µm, Q3: 3.5 µm) (SI Figure S3a). Neat PLLA scaffolds exhibited relatively smooth surfaces (Figure 2, OP image), yielding the average peak-to-valley roughness (*R_z_*) and the arithmetic mean roughness (*R_a_*) values of 45.8 ± 13.6 µm and 5.8 ± 1.7 µm, respectively (Figure 2a). Upon HAP incorporation (16.7% and 23.1% loadings), the scaffold morphology changed significantly: the fibers became thicker, adhesions formed between the fibers, the surfaces become rougher, and distinct HAP particles could be seen on the scaffold surface (Figure 2, SEM and OP images). After adding HAP, the median pore area of the prepared scaffolds increased by approximately two to three times (from 25.8 µm^2^ to (57.3–72.0) µm^2^) (SI Figure S3). Scaffold samples “16.7%”, “23.1%” containing HAP have approximately twice the median fiber diameter (SI Figure S3) and are three to four times rougher (Figure 2a) than PLLA scaffolds without HAP. Bright-field microscopy images revealed that the HAP particles are evenly distributed across the surface of the composite scaffold samples (SI Figure S4). The median size of the HAP particles was measured at 28.4 µm (Q1: 20.7 µm, Q3: 36.2 µm) and 23.9 µm (Q1: 19.3 µm, Q3: 29.2 µm) for the “16.7%” and “23.1%” samples, respectively (SI Figure S5d,e), while the median particle size of HAP itself was 26.3 µm (Q1: 21.4 µm, Q3: 31.0 µm) (SI Figure S5c). The areal density of the neat PLLA scaffold was 3.89 mg/cm^2^, whereas the PLLA/HAP scaffolds exhibited approximately twofold higher areal densities, ranging from 7.69 to 8.86 mg/cm^2^ (Table S1). The porosity of the PLLA scaffold was 82%, while the PLLA/HAP scaffolds showed slightly lower porosity values, ranging from 75% to 77% (Table S1).

SEM analysis revealed that HAP particles were not completely encapsulated within the polymer and remained mostly exposed to the surface of the prepared scaffolds. These results demonstrate that incorporating hydroxyapatite microparticles with a median size of 26.3 µm yields a nonwoven composite scaffold with surface-exposed HAP particles. The localization of HAP microparticles on the surface of polymer fibers can be attributed to hydrodynamic effects. Due to the high inertia of HAP particles, they cannot fully follow the accelerating liquid phase polymer jet, leading to velocity slip between the dispersed and continuous phases of HAP and polymer, respectively. Under strong extensional flow, this mismatch may induces radial migration of particles toward the jet periphery [45]. Moreover, the surface exposure of HAP microparticles is likely associated with the differences between size of HAP microparticles and the diameter of the constantly thinning polymer jet. When the diameter of thinning jet becomes less or comparable with diameter of HAP particles, the possibility of complete encapsulation of particles becomes less likely, leading to particle exposure on the fiber surface. These suggestions are supported by the finding that exposed HAP particles (SI Figure S5d,e) are substantially bigger in size than the median fiber diameter of composite PLLA/HAP scaffolds (SI Figure S3b,c).

The significant morphological changes observed upon the addition of HAP microparticles are specifically related to the increase in fiber diameter, pore size, and surface roughness. Similar findings were reported in [46], where the addition of HAP/SiHA microparticles (with aggregate size from ~2 to 124 µm) significantly changed the morphology of electrospun polyester scaffolds, which was associated with the formation of a beaded, rough surface morphology and larger fibers (shifting the predominant range from 1 to 5 µm to 5–10 µm). An increase in viscosity leads to an increase in the length of the straight jet region and reduces the whipping angle during bending instabilities [47]. Concurrently, the lower electrical conductivity stabilizes the straight jet while attenuating the stretching forces [48]. As a result, the decrease in conductivity and the increase in viscosity lead to a significant increase in the fiber diameter [47,49,50]. Furthermore, due to their lower surface-to-volume ratio, solvent evaporation from thicker fibers is slower, and the fibers can reach the collector in a partially wet state, which may lead to the formation of fused junctions between fibers [47].

The obtained morphology, in which hydroxyapatite particles remain partially exposed rather than fully embedded, is often considered more advantageous than internal encapsulation. Such surface-localized particles exhibit enhanced bioavailability, as they are directly accessible for interaction with physiological fluids, thereby promoting cell proliferation and differentiation [21,51]. Moreover, HAP particles in the polymer composite provide nucleation sites for apatite reprecipitation in simulated body fluid [52], enhancing bioactivity and accelerating scaffold integration with surrounding bone tissue.

An absorbance peak at a wavenumber of 1021 cm^−1^ indicates the P-O stretching of the phosphate –${PO}_{4}^{3-}$ group in HAP [53]. The position of this peak was evaluated based on the FTIR spectrum of hydroxyapatite powder (SI Figure S5b), which was used for the fabrication of PLLA/HAP composite scaffolds. An increase in the intensity of this region in an FTIR spectrum may indicate an increase in HAP content (Figure 2b). Peaks at wavenumbers 1081 cm^−1^ and 1181 cm^−1^ can be attributed to the C-O stretching vibration of PLLA [54]. The wavenumber of 1748 cm^−1^ can be associated with the stretching of the carbonyl group (C=O) in the PLLA polymer [55]. Peaks at a wavenumber of 1454 cm^−1^ and in the region of 2940–2990 cm^−1^ indicate the bending and stretching of the methyl group (CH_3_) in PLLA, respectively [56,57].

Elemental analysis using X-ray fluorescence (XRF) indicates that the atomic C/O ratio is 2.13 for PLLA and 2.01 for PLLA/HAP scaffolds (SI Figure S6a). This slight decrease in the C/O ratio is related to the presence of hydroxyapatite particles, which contain –OH and –${PO}_{4}^{3-}$ groups. The atomic ratio of Ca/P in PLLA/HAP scaffolds is 1.67–1.75, which is very close to the stoichiometric composition of hydroxyapatite, that has an atomic calcium to phosphorus ratio of 1.67 [58]. The energy dispersive X-ray spectroscopy (EDX) results demonstrate that the atomic ratios of C/O and Ca/P are equal to 2.28–2.34 and 1.86–1.91, respectively (SI Figure S6b). Such differences can be related to the different analyzed penetration depths and sensitivity of XRF and EDX methods [59].

The X-ray diffraction results demonstrate that the PLLA scaffold is amorphous, as only an amorphous halo is present at 2*θ* values in the range of 10° to 30° (Figure 2c). For the “16.7%” and “23.1%” samples, a crystalline peak of PLLA with a plane structure of (200/110) is observed at 16.8° [60], along with the most intense HAP peak corresponding to the (211) reflection at 31.9° (Figure 2c) [61]. PLLA/HAP scaffold with a lower content of HAP exhibits the most intense crystalline peak of PLLA. The phenomenon of PLLA crystalline peaks formation after the addition of HAP has been reported previously, where PLLA crystallization occurs due to the HAP particles acting as nucleation centers [62]. The crystallite size of PLLA polymer and HAP particles were calculated based on most intense crystallographic planes: (200/110) for PLLA and (211) for HAP. For the samples “16.7%” and “23.1%”, the crystallite size of PLLA were equal to 14.8 ± 0.5 nm, and 14.5 ± 0.8 nm, respectively (SI Table S2). The crystallite size of mineral phase with HAP were equal to 7.6 ± 0.4 nm and 6.7 ± 0.9 nm for the scaffolds “16.7%” and “23.1%”, respectively (SI Table S2). The crystallinity degrees of PLLA in the “16.7%” and “23.1%” scaffold samples were equal to 6.8 ± 0.4% and 3.7 ± 0.3%, respectively (SI Table S2). While the addition of HAP microparticles initially induces crystallization in the amorphous neat PLLA via heterogeneous nucleation, higher HAP loading led to a noticeable decrease in PLLA crystallinity. This trend can be attributed to increased particle agglomeration at elevated filler contents, which disrupts the polymer nucleation process [63]. Furthermore, HAP particles are known to reduce the growth rate of PLLA spherulites [64]. They act as physical barriers that prevent continuous crystallization and the growth of PLLA crystallites. As a result, at higher HAP concentrations, this physical restriction of crystal growth outweighs the initial nucleation effect, resulting in reduction in overall PLLA crystallinity.

After heating to 800 °C, neat PLLA decomposes completely, whereas in the “16.7%” and “23.1%” samples, residues remain with corresponding masses of 16.5 ± 1.3 wt.% (2.2 ± 0.2 mg) and 20.1 ± 1.3 wt.% (3.3 ± 0.4 mg), indicating the presence of residual HAP (Figure 2d and SI Figure S7a). The difference between the theoretical (23.1%) and experimentally determined (20.1%) mass of HAP for the “23.1%” sample is due to the fact that the PLLA/HAP dispersions applied for electrospinning are unstable and can be properly electrospun only after ultrasonication, since HAP slowly settles at the bottom of the syringe over time [65]. Nevertheless, the relatively low standard deviation of the residual masses of the PLLA/HAP scaffolds after thermal decomposition indicates the good reproducibility of the scaffold fabrication process.

The mass loss rate curve of the PLLA scaffolds indicates that decomposition begins at a temperature of approximately 310 °C, and the maximum mass loss temperature (T_max_) is observed at around 377 °C (SI Figure S7b). Similar results are reported in reference [66], where PLA decomposition is observed in the temperature range of 290–380 °C. In one systematic review, it was mentioned that polylactide is usually degraded in a single step, with the maximum mass loss rate reached at 350 °C [67]. The DTG results obtained confirm the single-step degradation of PLLA scaffolds (SI Figure S7b). PLLA/HAP scaffolds exhibit similar T_max_ values compared to neat PLLA samples. The absence of a shift indicates the retention of the chemical structure in the composite materials.

**Figure 3:**
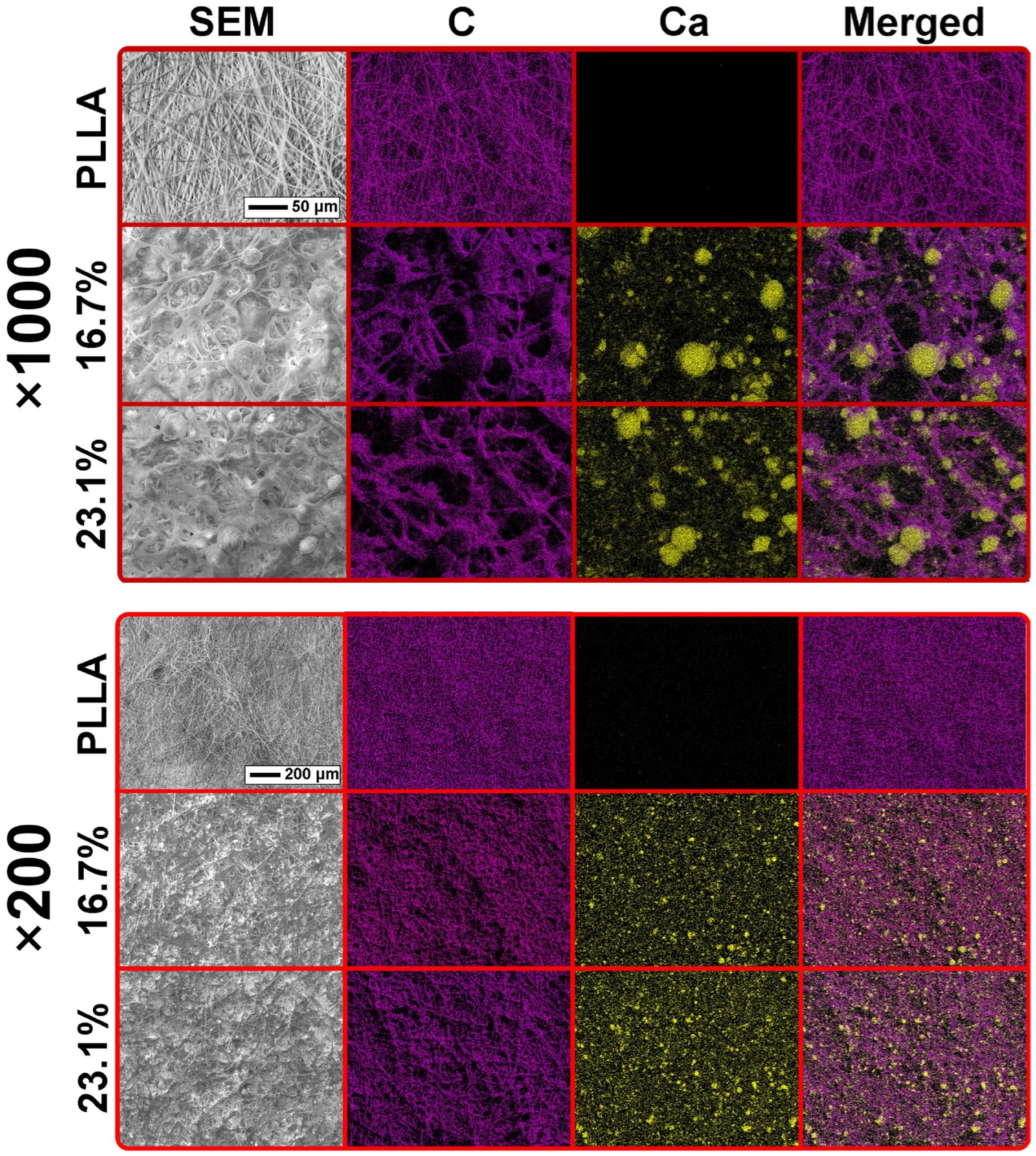
Scanning electron microscopy images (SEM) and corresponding energy dispersive X-ray (EDX) mapping images (for the elements: C—carbon, Ca—calcium, Merged—merged channels of carbon and calcium) at ×1000 magnifications (in the upper part of the Figure) and ×200 magnifications (in the lower part of the Figure).

SEM images and corresponding EDX mapping images at ×200 and ×1000 magnifications of the prepared PLLA and PLLA/HAP electrospun scaffolds are presented in Figure 3.

Nonwoven fibers from PLLA scaffolds without HAP consist only of carbon and oxygen (Figure 3 and SI Figure S8). PLLA scaffolds with HAP have a morphology, in which polymer fibrous matrix contain distinct spherical microparticles, that mainly consist of calcium, oxygen and phosphorus (Figure 3 and SI Figure S8). Signals of calcium channels from spherical HAP particles can be observed in the EDX mapping images with merged carbon and calcium channels (Figure 3). It is seen that the carbon signal does not overlap with the calcium signal. These results indicate that the biggest HAP particles on the matrix surface are mainly in exposed form, which is more clearly observed in SEM images acquired at ×1000 magnifications. Quantitative analysis at ×1000 magnifications demonstrated that the surface area occupied by HAP particles was 12.3 ± 0.9% and 14.2 ± 1.2% of the total scaffold surface area for the “16.7%” and “23.1%” samples, respectively (Table S3). The surface area occupied by HAP particles increased with increasing HAP content in the scaffolds, indicating a direct correlation between filler loading and surface coverage. Although theoretical studies support that PLLA/HAP affinity may promote anchoring of particles at the polymer interface [20,68], large size and low specific surface area of these microparticles restrict complete polymer coating, leaving partially exposed HAP (Figure 2 and Figure 3). At ×200 magnifications the HAP particles are mainly uniformly distributed across scaffold surface (Figure 3). Obtained EDX mapping data confirms previous SEM observations (Figure 2) that incorporation of relatively large HAP microparticles provides a successful strategy to create PLLA scaffolds with surface-exposed mineral phase particles.

Several studies have demonstrated that the immobilization of HAP particles on the scaffold surface enhances their biological activity by increasing direct contact with the surrounding environment, thereby promoting protein adsorption, cell attachment, proliferation, and osteogenic differentiation [21,51]. Furthermore, the incorporation of HAP into bioinert polymer scaffolds significantly improves apatite nucleation and growth upon immersion in SBF [69,70]. For example, apatite deposition on neat polyester scaffolds was negligible, but became pronounced after the addition of HAP particles, resulting in the formation on the scaffold surface of an apatite layer [69]. In addition, a positive correlation was established between the HAP content on the PLLA scaffold surfaces and the amount of deposited apatite from SBF solution on these scaffolds [71].

The quantitative analysis of HAP particles distribution at ×200 magnifications showed that the coefficient of variation, Gini coefficient and Global Moran’s I did not differ significantly between the PLLA/HAP scaffolds containing 16.7 wt.% and 23.1 wt.% HAP (SI Figure S9). This indicates that the distribution of HAP-containing regions was comparable for both scaffold types. However, all three parameters were highly sensitive to the image partitioning scheme used for the analysis. The EDX mapping images were divided into grids of 8 × 8, 16 × 16, 32 × 32, or 64 × 64 cells. As the number of partitions increased (i.e., as the grid cells became smaller), values of all three parameters increased. For example, coarse grids (8 × 8 and 16 × 16) yielded relatively low Gini coefficients (Gini < 0.5), indicating a nearly homogeneous distribution of HAP among the grid cells, while Global Moran’s I value close to zero (Moran’s I ≈ 0) indicated little or no spatial autocorrelation. In contrast, finer grids (32 × 32 and 64 × 64) produced higher Gini coefficients (Gini > 0.5) together with positive Moran’s I values, revealing greater local heterogeneity and increasingly pronounced spatial clustering. These findings indicate that the HAP particles are relatively uniformly distributed at larger spatial scales, whereas local clustering becomes noticeable at smaller scales. Such scale dependence is expected for heterogeneous particulate systems and has been reported in studies related with spatial distribution in case of different sampling unit resolution [72,73]. It is worth noting that this analysis does not provide any direct information about HAP particle agglomeration.

### 3.3. Mechanical Properties and Wettability of the PLLA and PLLA/HAP Scaffold Samples

**Figure 4:**
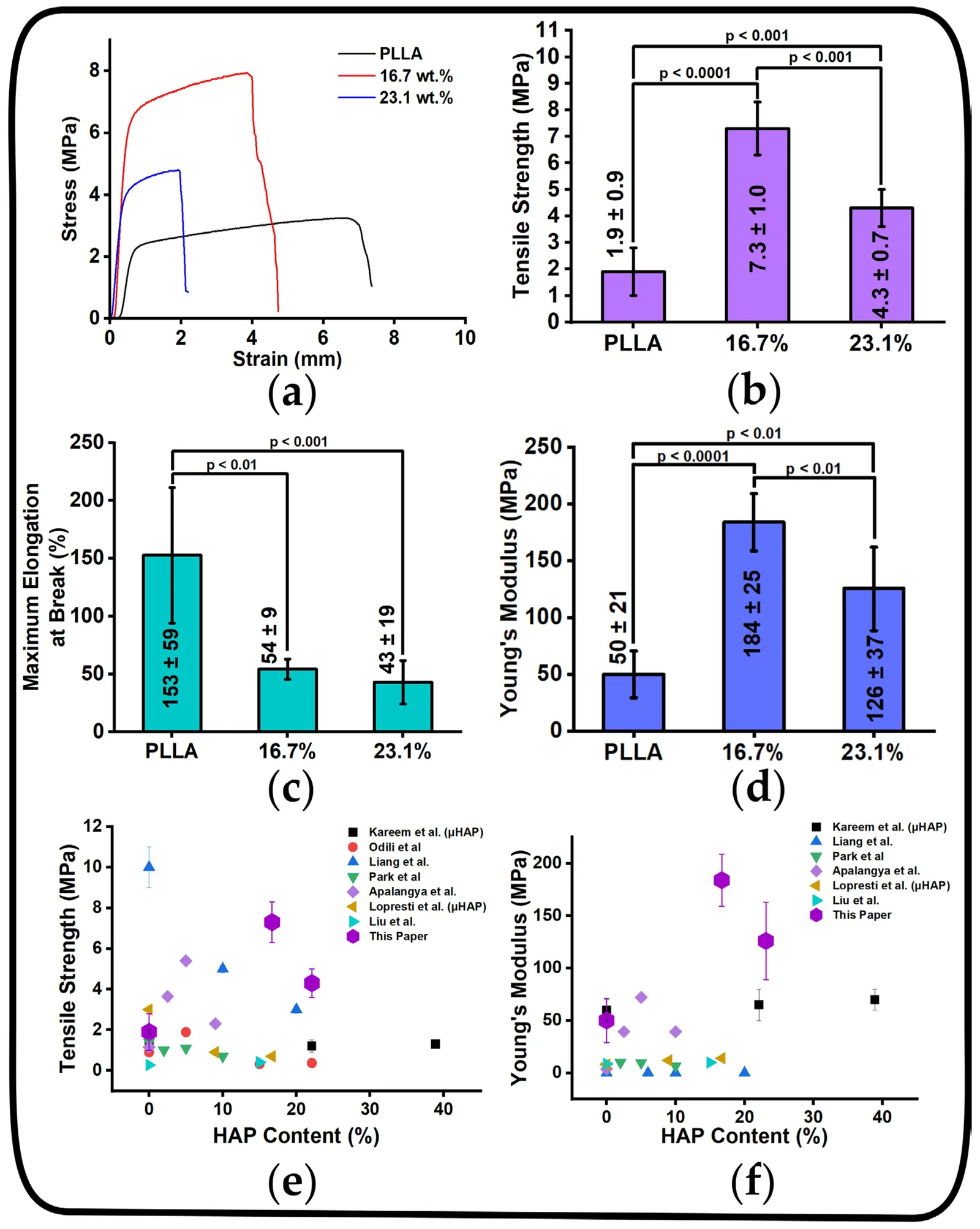
Mechanical properties of the prepared electrospun PLLA and PLLA/HAP scaffolds and their comparative analysis with literature data: (**a**) Stress–strain curves, (**b**) tensile strength values, (**c**) maximum elongation at break, (**d**) Young’s modulus (modulus of elasticity), comparative analysis of (**e**) tensile strength and (**f**) Young’s modulus for (PLLA or PLA)/HAP scaffolds, which were prepared in this study and those reported in other literature. Note: The literature data of tensile strength and Young’s modulus were gathered from the following references: Kareem et al. [27], Odili et al. [74], Liang et al. [25], Park et al. [75], Apalangya et al. [76], Lopresti et al. [24], and Liu et al. [77].

The mechanical properties of the prepared PLLA/HAP scaffolds as well as the comparative analysis of their tensile strength and Young’s modulus with literature data on electrospun polylactide/HAP scaffolds, are presented in Figure 4.

The stress–strain curves of the tested scaffolds (Figure 4a) exhibit the characteristic shape that is typically observed for electrospun composite polymer scaffolds based on aliphatic polyesters [78,79]. The tensile strength of PLLA scaffolds is 1.9 ± 0.9 MPa, while the HAP-containing scaffolds “16.7%” and “23.1%” exhibit substantially higher values of 7.3 ± 1.0 MPa and 4.3 ± 0.7 MPa, respectively (Figure 4b), which are 3.8-fold and 2.3-fold higher than that of neat PLLA. The PLLA scaffold demonstrates the highest maximum elongation at break, with a value of 153 ± 59%, while PLLA/HAP scaffolds increased their own length by approximately 43–54% until failure (Figure 4c). The Young’s modulus of PLLA scaffolds is 50 ± 21 MPa, whereas those values for the “16.7%” and “23.1%” scaffolds are approximately 3.7-fold and 2.5-fold higher, respectively (Figure 4d). In comparison with the previously reported studies, the prepared PLLA/HAP scaffolds mainly demonstrate noticeably higher tensile strength and Young’s modulus than the electrospun PLA/HAP and PLLA/HAP scaffolds described in the analyzed literature (Figure 4e,f).

High mechanical properties can be explained by the morphological features and XRD diffractograms of the prepared PLLA/HAP scaffolds. A significant increase in mechanical properties after addition of HAP microparticles is explained by a significant increase in fiber diameter and the formation of adhesions between fibers, which usually can be observed with an increase in solution viscosity [80]. In addition, Can-Herrera et al. reported that increasing the fiber diameter and forming adhesions between the fibers significantly increases the tensile strength of biodegradable electrospun polyesters [81]. Prepared composite scaffolds with a semicrystalline PLLA structure have the highest tensile strength values, while amorphous PLLA scaffolds exhibit the lowest tensile strength and the highest maximum elongation at break. Li et al. reported that higher crystallinity of electrospun PLLA scaffolds increases tensile strength and decreases maximum elongation at break [82]. A critical review article mentions that Young’s modulus of PLA increases with increasing crystallinity [83]. Crystallization in PLA results in enhanced stiffness and brittleness due to the reduced mobility of the polymer chains [83]. The mechanical and XRD results are in good agreement. The “16.7%” scaffold, which exhibits the highest PLLA crystallinity degree, also exhibits the best mechanical properties, whereas the “23.1%” scaffold, with a lower PLLA crystallinity degree, shows the lower mechanical performance. These results suggest that HAP particle agglomeration may occur in the “23.1%” scaffold. Such agglomeration can disturb PLLA chain ordering and crystal growth, while also acting as stress-concentration sites, thereby reducing both the crystallinity and mechanical properties of polymer-mineral composites [84].

The wettability results demonstrate that the water contact angle values did not change significantly after the addition of HAP particles and the water contact angle (WCA) for all samples was in the range of (116–124)° ± 5° (SI Figure S10a,b). The WCA values were constant during 1 min of droplet interaction with the surface of the prepared PLLA and PLLA/HAP scaffolds.

### 3.4. Biological and Osteogenic Properties of the PLLA and PLLA/HAP Scaffold Samples

**Figure 5:**
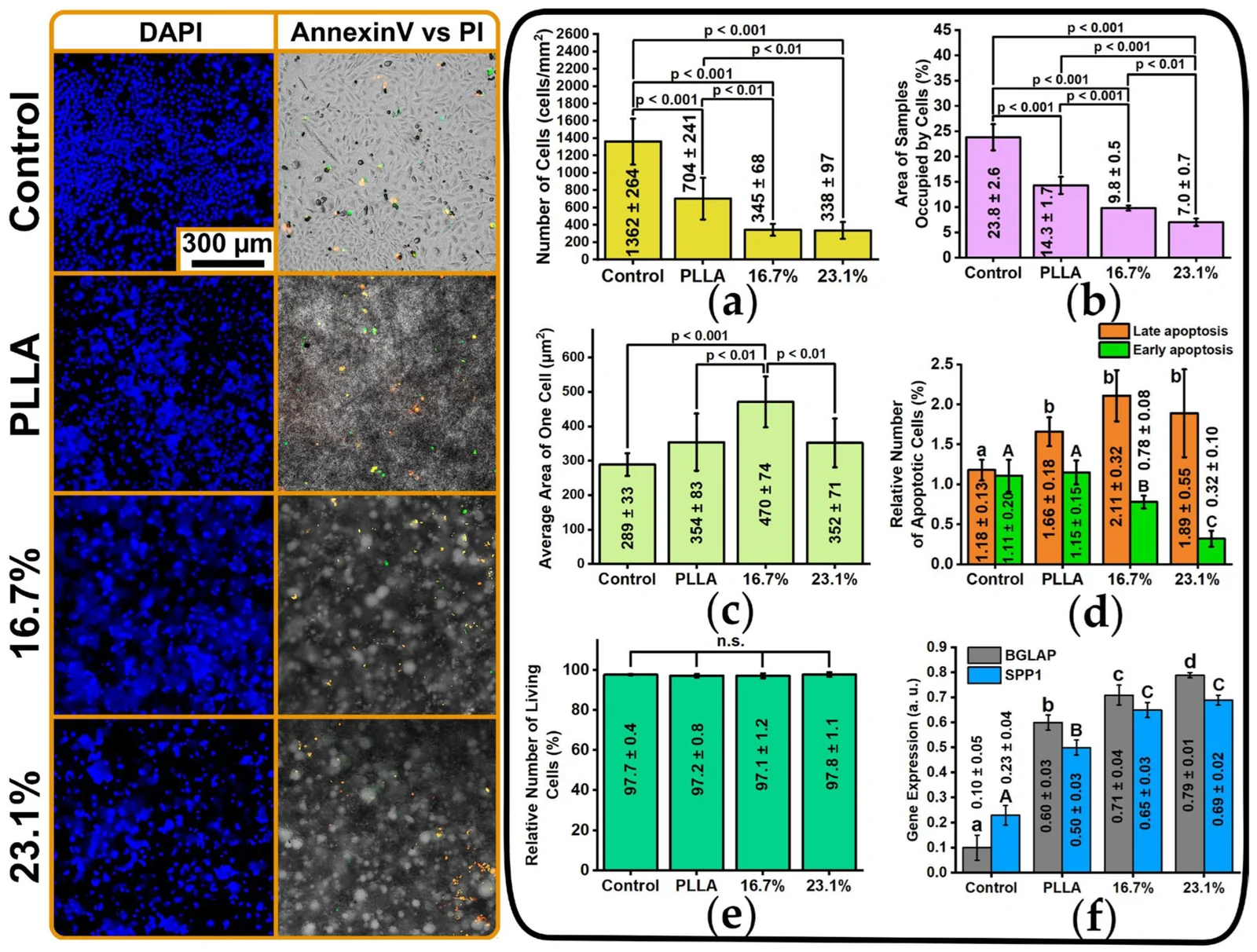
Biological and osteogenic properties of PLLA and PLLA/hydroxyapatite (HAP) scaffolds: (**a**) Number of human adipose-derived stromal/stem cells (hADSCs), (**b**) sample area occupied by hADSCs, (**c**) average area occupied by single hADSC, (**d**) relative number of apoptotic cells, (**e**) relative number of living cells, (**f**) gene expression results of osteocalcin (BGLAP) and osteopontin (SPP1). Note: Lowercase and uppercase letters in panels (**d**,**f**) denote separate sets of statistical comparisons, with different letters (e.g., a, b, c, d or A, B, C, D) indicating statistically significant differences (*p* < 0.05).

The biological and osteogenic properties of the prepared PLLA and PLLA/HAP scaffolds are presented in Figure 5.

According to the biological results, the highest number of human adipose-derived stromal/stem cells (hADSCs) with 1362 ± 264 cells/mm^2^ were found on the control samples (tissue culture plastic), while the number of cells on the PLLA scaffold samples is about half of that on the tissue culture plastic (704 ± 241 cells/mm^2^). On the samples “16.7%”, “23.1%” (with HAP), the number of cells is about four times lower than on the control samples (338–345 cells/mm^2^) (Figure 5a). The same dependence can be clearly observed in Figure 5b hADSCs-occupied sample area: the maximum cell area covers tissue culture plastic, while the smallest area occupied by cells is found for the “23.1%” PLLA/HAP scaffold samples. Nevertheless, the estimated average area values of one cell on scaffold samples demonstrated that the cells cultured on the PLLA/HAP scaffold containing 16.7 wt.% HAP exhibited a larger projected area (470 ± 74 µm^2^), compared with the tissue culture plastic control (289 ± 33 µm^2^), the neat PLLA scaffold (354 ± 83 µm^2^) and the PLLA/HAP scaffold containing 23.1 wt.% HAP (352 ± 71 µm^2^). In [85], it was demonstrated that the hADSCs size was equal to 10 µm (corresponding to a projected area of about ~78.5 µm^2^ if approximated as a circle). These results suggest that the cells on all scaffolds were well spread rather than exhibiting a rounded morphology. The percentage of apoptotic cells for all samples is in the range of approximately (2–3)% (the percentage of living cells is about (97–98)%), which indicates that none of the scaffolds are cytotoxic to hADSCs (Figure 5d,e). This decline in cell number and occupied area for PLLA/HAP scaffolds can be explained by the fact that they are rougher, have larger pore areas, and have a more developed surface than neat PLLA scaffolds and the tissue culture plastic (Figure 2). Such a morphology accelerates cell migration through the internal scaffold structure. As roughness increases, the rate of cell migration increases [86]. The review article [87] states that the pore size of electrospun scaffolds promotes cell migration. Murphy et al. report that polymer scaffolds with the largest pore size (325 µm) are more suitable for the migration and proliferation of MC3T3-E1 cells [88]. The relatively large size of HAP microparticles cannot be the issue either, as the cytotoxicity of HAP increases with decreasing size of the HAP particles [89,90]. Reference [89] reports that nanoscale HAP suppresses the viability and migration of endothelial cells, as nano-HAP can accumulate in the cell cytoplasm, whereas microscale HAP has no such effect. Furthermore, due to their proliferative and biological properties in vitro, HAP microparticles are preferable to HAP nanoparticles. Reference [91] reports that PLLA/HAP scaffolds with HAP microparticles and with random morphology have better cell viability and osteogenic properties than PLLA/HAP scaffolds with HAP nanoparticles.

Nevertheless, abovementioned explanations tend to be logical, the decrease in number of cells may be related to other possible factors. Excessive roughness and hydrophobicity of scaffolds surface can reduce the proliferative activity of osteoblasts and bone marrow-derived cells [92,93]. For example, rough and highly hydrophobic poly(L-lactic acid) surfaces showed lower attachment and adhesion of bone marrow-derived cells than smoother and more hydrophilic PLLA surfaces [93]. In addition, the hydroxyapatite-induced osteogenic differentiation of osteoblast cells may be accompanied by their reduced proliferation [94]. Moreover, hydrophobic surfaces generally alter the conformation of adhesive proteins after their secretion by cells, thereby limiting cell adhesion [95,96]. Cells do not attach directly to the polymer surface. Instead, after immersion in serum-containing culture medium, the scaffold surface rapidly becomes coated with adsorbed serum proteins that mediate cell adhesion. The conformation of these adsorbed proteins is influenced by surface wettability, and highly hydrophobic surfaces may reduce the availability of fibrinogen sites for cells [96]. However, there are also studies demonstrating that mesenchymal cells can successfully attach to highly hydrophobic surfaces [97], indicating that surface wettability alone does not determine cell adhesion and that other factors, including morphology, surface chemistry and roughness also play important roles. In addition, several studies have reported that hydroxyapatite incorporation improves protein adsorption on poorly wettable surfaces of PLLA and PLA [98,99,100].

The nonwoven structure of the PLLA scaffold stimulates gene expression in hADSCs: BGLAP expression increased sixfold and SPP1 expression increased approximately twofold compared to the control tissue culture plastic (Figure 5f). Such a fibrous morphology of biodegradable polyesters has a positive effect on the differentiation of hADSCs [101]. The addition of HAP into the scaffolds increases the expression of BGLAP and SPP1 by approximately 1.2–1.4-fold relative to the PLLA scaffolds (Figure 5f). hADSCs cultured on HAP-containing scaffolds exhibit BGLAP and SPP1 expression levels that are 7.1–7.9-fold and 2.8–3.0-fold higher, respectively, than those observed on control tissue culture plastic. These results may confirm the positive effect of the obtained morphology of composite PLLA/HAP scaffolds, containing HAP in exposed form on the osteogenic differentiation of cells.

It can be concluded that the exposed Ca/P-rich HAP domains observed by SEM/EDX may be a plausible basis for the moderate enhancement of osteogenic marker expression. Such results are consistent with previous research. Cai et al. demonstrated that HAP particles embedded beneath the polymer surface are not fully exploited by cells, whereas surface-exposed HAP noticeably improves MC3T3-E1 attachment, proliferation, and osteogenic differentiation [21]. This supports the idea that HAP accessibility can be a key factor governing the bioactivity of polymer/HAP composites. As an example, consider a study demonstrating the importance of HAP availability: in reference [102], it was demonstrated that the addition of HAP slightly increases osteocalcin expression for electrospun PLLA/PCL scaffolds, but such an increase was not statistically significant from the control group. This may be due to the absence of exposed HAP particles.

### 3.5. Mechanism of Fiber Formation

**Figure 6:**
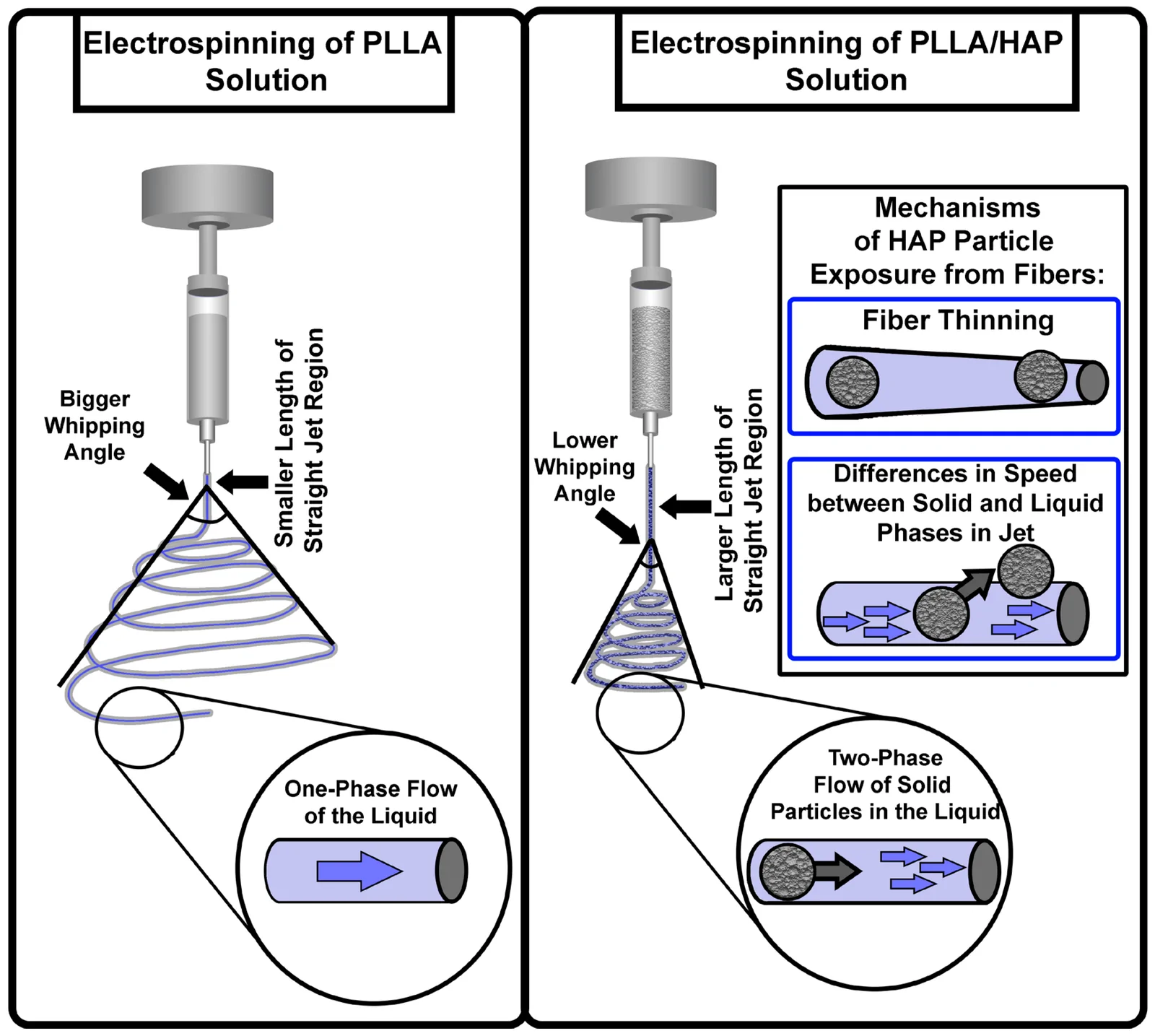
Proposed mechanisms of PLLA and PLLA/HAP jet and fiber formation during electrospinning: panel on the left—PLLA jet and fiber formation process; panel on the right—PLLA/HAP jet and the fiber formation mechanism with the presence of HAP microparticles. Note: Blue and gray arrows inside the polymer jet indicate the directions of polymer solution and HAP particle movement, respectively.

The mechanism of PLLA fiber formation with HAP microparticles and their comparison with the neat PLLA fiber formation process using an electrospinning method are presented in Figure 6.

Formation of PLLA fibers using an electrospinning setup, in which the spinning solution contains only one liquid phase (polymer/solvent), involves the following processes: the occurrence of bending instabilities, during which the polymer jet becomes thinner [103], and simultaneous jet solidification caused by solvent evaporation [104]. In that case, the polymer and solvent phases behave as a single fluid with a common velocity (Figure 6). As a result, smooth fibers from solutions with one liquid phase are usually fabricated during the electrospinning process [105]. The length of the straight jet region tends to decrease with decreasing solution viscosity of the spinning solution [106]. The relatively low viscosity of the polymer solution makes it possible to initiate bending instabilities earlier with a higher whipping angle of the bending jet, which tends to the formation of thin fibers [47]. Due to their higher surface area to volume ratio, thinner fiber can solidify more easily and quickly than thicker ones due to faster solvent evaporation. This promotes formation of nonwoven scaffolds with classical morphology, characterized by the absence of fiber fusion and melted junctions with a relatively narrow fiber diameter distribution (Figure 2 and SI Figure S3a).

The electrospinning of suspensions containing both liquid and solid phases can differ substantially from the electrospinning of homogeneous polymer solutions with one liquid phase. Large solid hydroxyapatite microparticles may not fully follow the rapid elongation and thinning of the polymer jet because of their size and higher density [45]. As a result, heavy particles may migrate toward surface of the jet (Figure 6). In addition, during jet thinning, some particles can be exposed when their size or the size of their clusters becomes comparable to or larger than the diameter of the formed fibers (Figure 2). The ability of an electrospun jet to carry particles whose size exceeds the diameter of the resulting fibers without jet stream disruption is governed by the viscoelastic properties of the polymer solution [3] and the polymer chain entanglement [107]. During jet elongation, the highly entangled polymer chains in the jet create partial or continuous envelopes around the HAP microparticles, allowing the jet to incapsulate relatively large inclusions without its disruption. In the present study, such envelopes are predominantly located beneath the HAP particles, while the upper surface of the HAP particles remains mostly exposed, especially for larger ones. That can be confirmed by SEM images and EDX mapping results (Figure 2 and Figure 3). As a result, the electrospinning process enables the formation of continuous polymer fibers with HAP inclusions whose size is substantially larger than the thickness of the fiber itself [108,109]. The addition of HAP increased the viscosity and lowered the conductivity of the spinning solution (SI Figure S2). These changes usually tend to increase the length of the straight jet region, lower the whipping angle and decrease the stretching possibilities of the jet [47,48]. As a result, the fiber diameter values become larger and the fiber diameter distribution becomes significantly broader (SI Figure S3b,c).

Such differences in the jet behavior between PLLA and PLLA/HAP solutions were demonstrated in video recordings of the electrospinning of these solutions via NANON-01A setup (SI Videos S1–S3). The video recordings demonstrate that PLLA solutions have a shorter straight jet region and a larger whipping angle, while PLLA/HAP solutions have longer straight jet region and a narrower whipping angle.

Consequently, in this study, HAP microparticles act as a component that substantially alters jet behavior during electrospinning, thereby determining the final morphology and structure of the resulting scaffold. The incorporation of HAP particles increases the fiber diameter, pore area, surface roughness, and positively contributes to a crystallinity degree of the PLLA polymer. Collectively, these structural changes lead to the enhanced mechanical properties of the composite PLLA/HAP scaffolds. In addition, the partial exposure of HAP particles on the surface of the scaffold may promote the osteogenic differentiation of hADSCs.

## 4. Conclusions

There remains a challenge in the development of electrospun poly(L-lactide)/hydroxyapatite (PLLA/HAP) composite scaffolds. Although the incorporation of hydroxyapatite into the scaffolds improves their biological properties, it typically compromises the mechanical integrity due to particle agglomeration. Furthermore, when hydroxyapatite particles are embedded in fibers or coated with polymer, they may not contribute to increasing the biological activity of the scaffold due to the lack of direct contact between the particles and physiological fluids and cells. These limitations hinder the development of PLLA/HAP composite scaffolds that simultaneously provide high mechanical properties and keep bioactive HAP particles accessible to cells.

In the scope of this work, PLLA/HAP scaffolds with surface-exposed HAP particles and superior mechanical properties were successfully fabricated. The results of this study demonstrate that the addition of hydroxyapatite microparticles to the poly-L-lactide spinning solution results in the formation of composite scaffolds with higher tensile strength and Young’s modulus than those of neat PLLA scaffolds. The improvement in mechanical properties is attributed to changes in morphology and polymer crystallinity. While the neat PLLA scaffold is amorphous, the composite PLLA/HAP scaffolds are semicrystalline. In addition, PLLA/HAP scaffolds consist of thicker fibers with fused junctions between them, while hydroxyapatite microparticles are partially exposed on the surface of those scaffolds. In contrast, the fibers of neat PLLA scaffold are thinner and without fused fiber junctions. The PLLA/HAP scaffolds also exhibit a more pronounced osteogenic response of hADSCs than the neat PLLA scaffold and control tissue culture plastic. The expression of osteogenic markers osteocalcin (BGLAP) and osteopontin (SPP1) was 1.2–1.4-fold higher on PLLA/HAP scaffolds than on neat PLLA. Compared with the tissue culture plastic, the expression levels of BGLAP and SPP1 on PLLA/HAP scaffolds were 7.1–7.9-fold and 2.8–3.0-fold higher, respectively. A mechanism for the fiber formation based on PLLA and PLLA/HAP scaffolds was proposed to explain the significant differences in morphology. The proposed mechanism relates the effect of the inclusion of HAP particles into the spinning solutions, which leads to subsequent changes in electrical conductivity and viscosity, to the behavior of the polymer jet during electrospinning. Overall, the obtained results demonstrate that composite polymer scaffolds containing HAP microparticles represent a promising alternative to classical nonwoven electrospun scaffolds applied for bone tissue engineering. Nevertheless, further studies, including additional osteogenic and bioavailability assays, as well as in vivo investigations of biocompatibility, bioactivity, and bone regeneration, are required to validate their potential for clinical application.

## 5. Patents

Arsalan D. Badaraev and Sergei I. Tverdokhlebov are associated with the intellectual property related to this work, including patents RU2836608C1 and RU2845253C1.

## Data Availability

The raw data supporting the conclusions of this article will be made available by the authors on request.

## Supplementary Materials

The following supporting information can be downloaded at: https://www.mdpi.com/article/10.3390/polym18162001/s1, Figure S1: Protocol of the Biomedical Ethics Committee of the Cancer Research Institute, Tomsk National Research Medical Center, Russian Academy of Sciences, confirming compliance with bioethical standards; Figure S2: Electrical conductivity and viscosity of PLLA and PLLA/HAP spinning solutions: (a) Electrical conductivity; (b) Viscosity. Note: “16.7%” refers to the PLLA/HAP spinning solutions containing 20 wt.% HAP relative to the PLLA mass, and “23.1%” refers to the PLLA/HAP solutions containing 30 wt.% HAP relative to the PLLA mass; Figure S3: Distribution histograms of fiber diameter and pore area of the prepared scaffolds: (a), (d) PLLA scaffolds; (b), (e) 16.7% PLLA/HAP scaffolds; (c), (f) 23.1% PLLA/HAP scaffolds, respectively, and box plots of (g) fiber diameter and (h) pore area values of the prepared scaffolds. Note: “16.7%” refers to the electrospun PLLA/HAP scaffolds containing 20 wt.% HAP relative to the PLLA mass, and “23.1%” refers to the electrospun PLLA/HAP scaffolds containing 30 wt.% HAP relative to the PLLA mass; Figure S4: Bright-field microscopy (BFM) images of PLLA and PLLA/HAP scaffolds at ×4 and ×10 magnification. Note: “16.7%” refers to the electrospun PLLA/HAP scaffolds containing 20 wt.% HAP relative to the PLLA mass, and “23.1%” refers to the electrospun PLLA/HAP scaffolds containing 30 wt.% HAP relative to the PLLA mass; Figure S5: (a) Bright-field microscope (BFM) images of hydroxyapatite (HAP) particles at ×10 magnification, and (b) Fourier Infrared (FTIR) spectrum of HAP particles, (c) distribution histogram of HAP particle size gathered from BFM images, (d) distribution histogram of HAP particle size in 16.7% scaffolds gathered from SEM images, (e) distribution histogram of HAP particle size in 23.1% scaffolds gathered from SEM images. Note: Histograms of hydroxyapatite particles were collected from BFM images at ×10 magnification, “16.7%” refers to the electrospun PLLA/HAP scaffolds containing 20 wt.% HAP relative to the PLLA mass, and “23.1%” refers to the electrospun PLLA/HAP scaffolds containing 30 wt.% HAP relative to the PLLA mass; Figure S6: (a) X-ray photoelectron fluorescence (XRF) results for the elements: C—carbon, O—oxygen, Ca—calcium, P—phosphorus with the listed C/O and Ca/ P ratios, (b) Energy dispersive X-ray spectroscopy (EDX) spectroscopy results for the elements: C—carbon, O—oxygen, Ca—calcium, P—phosphorus with the listed C/O and Ca/P ratios. Note: “16.7%” refers to the electrospun PLLA/HAP scaffolds containing 20 wt.% HAP relative to the PLLA mass, and “23.1%” refers to the electrospun PLLA/HAP scaffolds containing 30 wt.% HAP relative to the PLLA mass; Figure S7: Thermogravimetric analysis results for PLLA and PLLA/HAP scaffolds: (a) Thermogravimetric curves displaying mass loss in terms of weight as a function of temperature, (b) differential thermogravimetric curves representing the rate of mass loss as a function of temperature. Note: “16.7%” refers to the electrospun PLLA/HAP scaffolds containing 20 wt.% HAP relative to the PLLA mass, and “23.1%” refers to the electrospun PLLA/HAP scaffolds containing 30 wt.% HAP relative to the PLLA mass; Figure S8: EDX mapping images (for the elements: C—carbon, O—oxygen, Ca—calcium, P—phosphorus) of the PLLA and PLLA/HAP scaffold samples examined, taken at ×1000 and ×200 magnifications. Note: “16.7%” refers to the electrospun PLLA/HAP scaffolds containing 20 wt.% HAP relative to the PLLA mass, and “23.1%” refers to the electrospun PLLA/HAP scaffolds containing 30 wt.% HAP relative to the PLLA mass; Figure S9: Quantitative assessment of hydroxyapatite particle distribution based on EDX mapping images of PLLA/HAP scaffolds acquired at ×200 magnification using different image partitioning schemes (grid sizes: 8 × 8, 16 × 16, 32 × 32, and 64 × 64): (a) coefficient of variation (%), (b) Gini coefficient, and (c) Global Moran’s I. Note: The hydroxyapatite particle size distributions were obtained from BFM images acquired at ×10 magnification. “16.7%” denotes electrospun PLLA/HAP scaffolds containing 20 wt.% HAP relative to the PLLA mass, whereas “23.1%” denotes electrospun PLLA/HAP scaffolds containing 30 wt.% HAP relative to the PLLA mass; Figure S10. Wettability of PLLA and PLLA/HAP scaffolds: (a) digital photographs of water droplets after 1 min of interaction with the scaffold surface, (b) water contact angles after 2 s and 1 min of interaction of water droplets with surface of the scaffolds. Note: “16.7%” refers to the electrospun PLLA/HAP scaffolds containing 20 wt.% HAP relative to the PLLA mass, and “23.1%” refers to the electrospun PLLA/HAP scaffolds containing 30 wt.% HAP relative to the PLLA mass. Table S1: Areal density and porosity of PLLA and PLLA/HAP scaffolds calculated via gravimetric method. Table S2: Crystallite size and crystallinity degree for the prepared PLLA/HAP scaffold samples, which was estimated based on X-ray diffractograms. Table S3: The relative area occupied by HAP particles on the surface of PLLA/HAP scaffolds, calculated using SEM and EDX mapping images. Video S1: Jet behavior of PLLA solution. Video S2: Jet behavior of 20% HAP solution. Video S3: Jet behavior of 30% HAP solution.
