# Supplementary material for: Surface-Exposed Hydroxyapatite Microparticles in Electrospun PLLA Scaffolds: Mechanical Reinforcement and Osteogenic Response
Source: Polymers (Basel). 2026 Aug 17;18(16):2001. doi: 10.3390/polym18162001 (PMC13516627; doi:10.3390/polym18162001)
Supplement: Supplementary file 1 [file polymers-18-02001-s001.zip › polymers-4451364-supplementary.pdf]

Supplementary Information of: Surface-Exposed Hydroxyapatite Microparticles in Electrospun PLLA Scaffolds: Mechanical Reinforcement and Osteogenic Response

Arsalan D. Badaraev [1,2], Mikhail A. Buldakov [3], Vladislav R. Bukal [2], Evgeny L. Choinzonov [3], Sven Rutkowski [2], Xiaojun Han [1,*] and Sergei I. Tverdokhlebov [2,*]

[1] State Key Laboratory of Urban-Rural Water Resource and Environment, Heilongjiang Provincial Joint Laboratory of Molecular Science (International Cooperation), School of Chemistry and Chemical Engineering, Harbin Institute of Technology, Harbin 150001, China; adb6@tpu.ru

[2] Weinberg Research Center, School of Nuclear Science & Engineering, National Research Tomsk Polytechnic University, 30, Lenin Avenue, 634050 Tomsk, Russia; vrb2@tpu.ru (V.R.B.); sven-rut@gmx.de (S.R.)

[3] Cancer Research Institute, Tomsk National Research Medical Center, Russian Academy of Sciences, 5 Per. Kooperativny, 634050 Tomsk, Russia; buldakov@oncology.tomsk.ru (M.A.B.); choynzonov@tnimc.ru (E.L.C.)

* Correspondence: hanxiaojun@hit.edu.cn (X.H.); tverd@tpu.ru (S.I.T.)

EXTRACT FROM A PROTOCOL 11

Meeting of the Biomedical Ethics Committee Cancer Research Institute, Tomsk National Research Medical Center, Russian Academy of Sciences, Tomsk, Russia
23 October 2015

Committee Chair – M.V. Vusik, Doctor of Medical Sciences
Committee Deputy Chair – T.L. Kravchuk, Doctor of Medical Sciences

6 committee members were present and cconsidered of the issue of compliance with bioethical standards creation of depositaries of biological samples and materials "BioBank"
Supervisor: N.N. Babyshkina
Investigator: E.A. Malinovskaya

Submitted documents:
1. Samples of Informed Consent from patients to participate in a scientific study in accordance with Articles 30, 31, 32, 33 of the "Basic Legislation on the Protection of Citizens' Health" dated 07/22/93 No.5487-1;
2. Application addressed to the Chairman of the Biomedical Ethics Committee, MD, M.V. Vusik, describing the research method on the specified topic;
3. Order 11 of 30.01.12 on the creation depositaries of biological samples and materials "BioBank" in Cancer Research Institute;
4. Regulations of depositary of biological samples and materials

RESOLVED:

To approve approve the submitted information consents, since the documents were prepared in accordance with legal requirements of Russian Federation and in accordance with the principles regulated by the Declaration of Helsinki of the World Medical Association.

Committee Chair – M.V. Vusik

Figure S1. Protocol of the Biomedical Ethics Committee of the Cancer Research Institute, Tomsk National Research Medical Center, Russian Academy of Sciences, confirming compliance with bioethical standards.

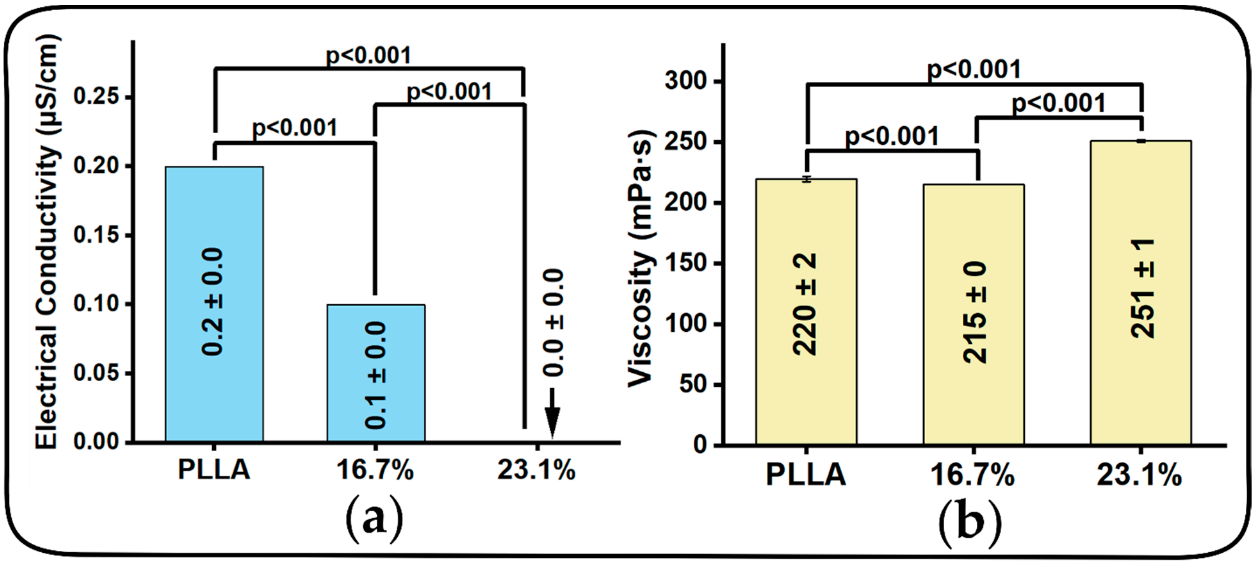

Figure S2. Electrical conductivity and viscosity of PLLA and PLLA/HAP spinning solutions: (a) Electrical conductivity; (b) Viscosity. Note: "16.7%" refers to the PLLA/HAP spinning solutions containing 20 wt.% HAP relative to the PLLA mass, and "23.1%" refers to the PLLA/HAP solutions containing 30 wt.% HAP relative to the PLLA mass.

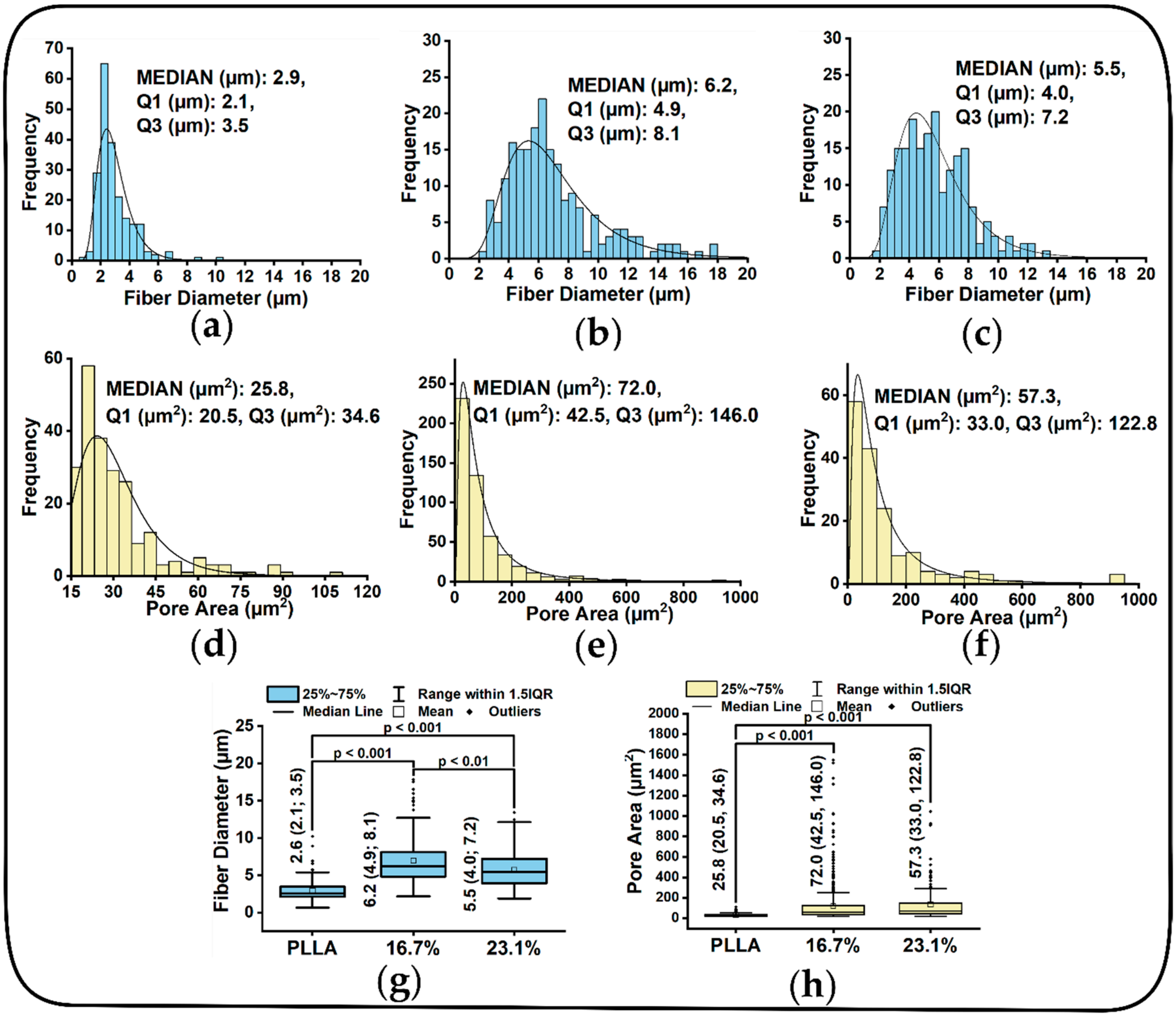

Figure S3. Distribution histograms of fiber diameter and pore area of the prepared scaffolds: (a), (d) PLLA scaffolds; (b), (e) 16.7% PLLA/HAP scaffolds; (c), (f) 23.1% PLLA/HAP scaffolds, respectively, and box plots of (g) fiber diameter and (h) pore area values of the prepared scaffolds. Note: "16.7%" refers to the electrospun PLLA/HAP scaffolds containing 20 wt.% HAP relative to the PLLA mass, and "23.1%" refers to the electrospun PLLA/HAP scaffolds containing 30 wt.% HAP relative to the PLLA mass.

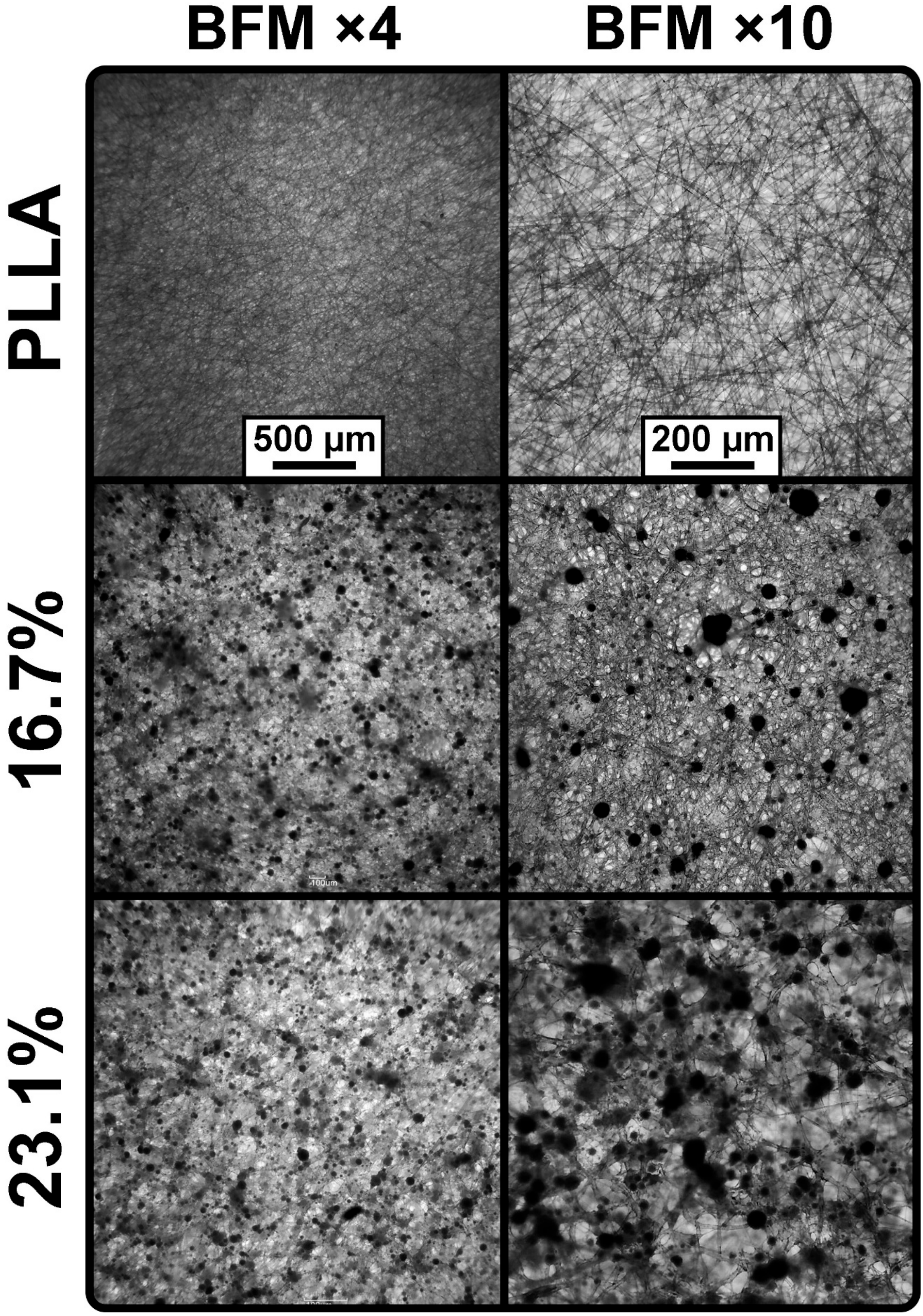

Figure S4. Bright-field microscopy (BFM) images of PLLA and PLLA/HAP scaffolds at ×4 and ×10 magnification. Note: "16.7%" refers to the electrospun PLLA/HAP scaffolds containing 20 wt.% HAP relative to the PLLA mass, and "23.1%" refers to the electrospun PLLA/HAP scaffolds containing 30 wt.% HAP relative to the PLLA mass.

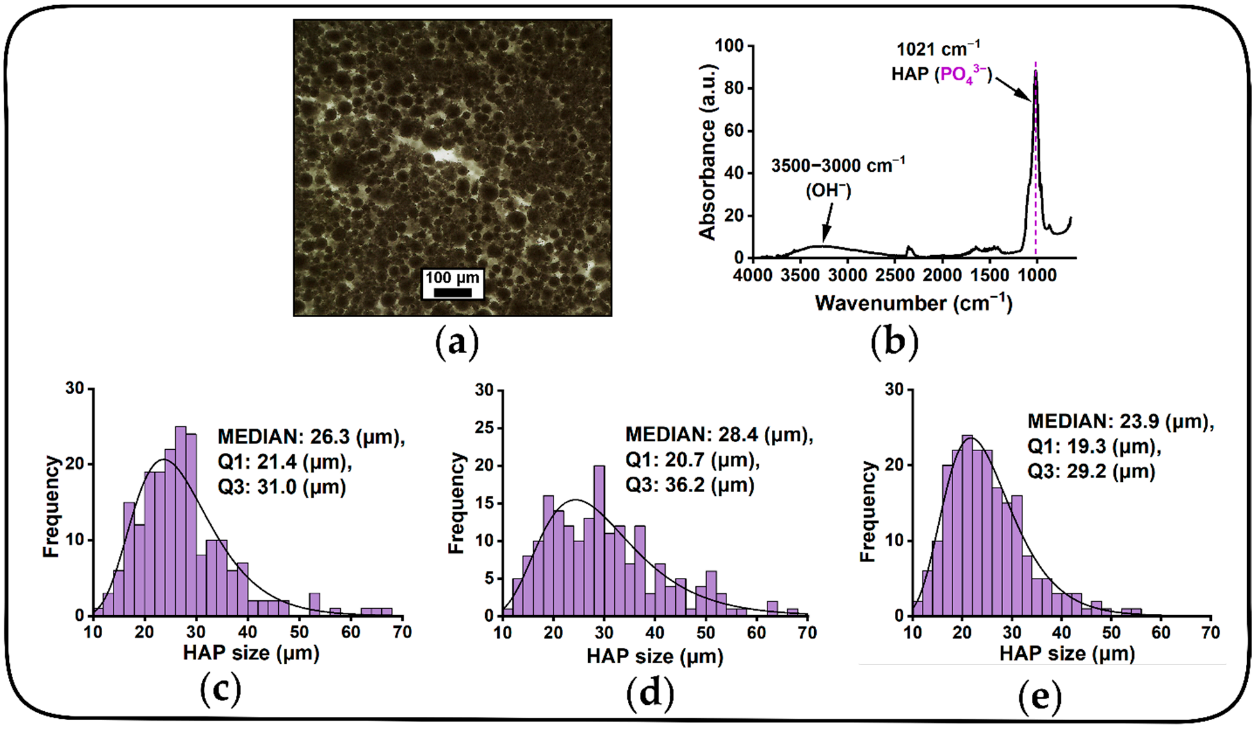

Figure S5. (a) Bright-field microscope (BFM) images of hydroxyapatite (HAP) particles at ×10 magnification, and (b) Fourier Infrared (FTIR) spectrum of HAP particles, (c) distribution histogram of HAP particle size gathered from BFM images, (d) distribution histogram of HAP particle size in 16.7% scaffolds gathered from SEM images, (e) distribution histogram of HAP particle size in 23.1% scaffolds gathered from SEM images. Note: Histograms of hydroxyapatite particles were collected from BFM images at ×10 magnification, "16.7%" refers to the electrospun PLLA/HAP scaffolds containing 20 wt.% HAP relative to the PLLA mass, and "23.1%" refers to the electrospun PLLA/HAP scaffolds containing 30 wt.% HAP relative to the PLLA mass.

XRF

	PLLA		16.7%		23.1%	
	at. %	wt. %	at. %	wt. %	at. %	wt. %
C	68.1 ± 1.8	61.6 ± 2.7	64.6 ± 0.2	54.9 ± 0.3	64.1 ± 0.1	53.9 ± 0.1
O	31.9 ± 0.8	38.4 ± 2.7	32.1 ± 0.1	36.3 ± 0.4	31.9 ± 0.3	35.7 ± 0.4
Ca	-	-	2.1 ± 0.1	6.1 ± 0.2	2.5 ± 0.1	7.2 ± 0.3
P	-	-	1.2 ± 0.1	2.7 ± 0.2	1.5 ± 0.1	3.2 ± 0.2
C/O ratio	2.13	1.60	2.01	1.51	2.01	1.51
Ca/P ratio	-	-	1.75	2.26	1.67	2.25

(a)

EDX

	PLLA		16.7%		23.1%	
	at. %	wt. %	at. %	wt. %	at. %	wt. %
C	69.5 ± 0,4	63.0 ± 0,4	67.8 ± 0.1	58.2 ± 0.2	67.2 ± 0.4	57.0 ± 0.7
O	30.5 ± 0,5	37.0 ± 0,7	29.0 ± 0.1	33.2 ± 0.1	28.8 ± 0.1	32.5 ± 0.2
Ca	-	-	2.1 ± 0.1	6.1 ± 0.3	2.6 ± 0.2	7.5 ± 0.6
P	-	-	1.1 ± 0.1	2.5 ± 0.1	1.4 ± 0.1	3.0 ± 0.2
C/O ratio	2.28	1.70	2.34	1.75	2.33	1.75
Ca/P ratio	-	-	1.91	2.44	1.86	2.50

(b)

Figure S6. (a) X-ray photoelectron fluorescence (XRF) results for the elements: C – carbon, O – oxygen, Ca – calcium, P – phosphorus with the listed C/O and Ca/ P ratios, (b) Energy dispersive X-ray spectroscopy (EDX) spectroscopy results for the elements: C – carbon, O – oxygen, Ca – calcium, P – phosphorus with the listed C/O and Ca/P ratios. Note: "16.7%" refers to the electrospun PLLA/HAP scaffolds containing 20 wt.% HAP relative to the PLLA mass, and "23.1%" refers to the electrospun PLLA/HAP scaffolds containing 30 wt.% HAP relative to the PLLA mass.

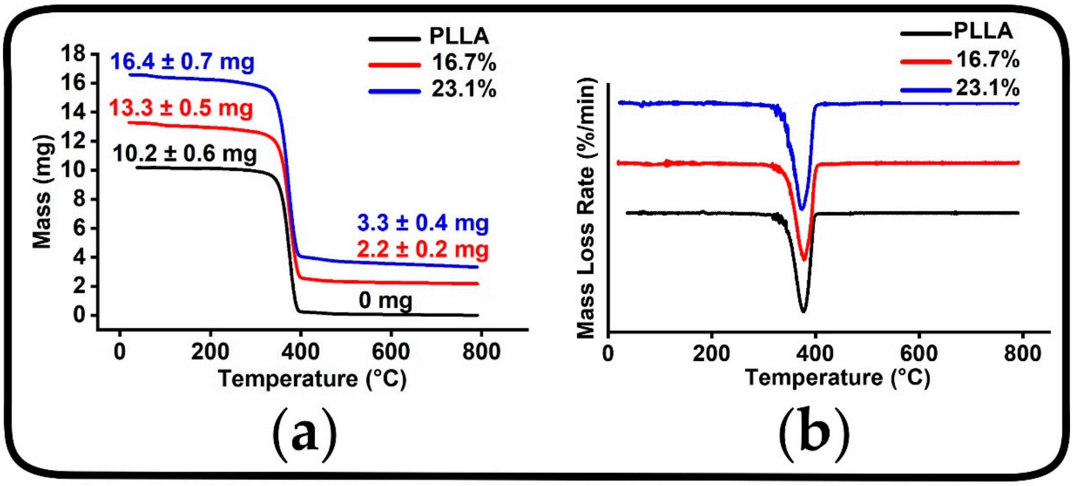

Figure S7. Thermogravimetric analysis results for PLLA and PLLA/HAP scaffolds: (a) Thermogravimetric curves displaying mass loss in terms of weight as a function of temperature, (b) differential thermogravimetric curves representing the rate of mass loss as a function of temperature. Note: "16.7%" refers to the electrospun PLLA/HAP scaffolds containing 20 wt.% HAP relative to the PLLA mass, and "23.1%" refers to the electrospun PLLA/HAP scaffolds containing 30 wt.% HAP relative to the PLLA mass.

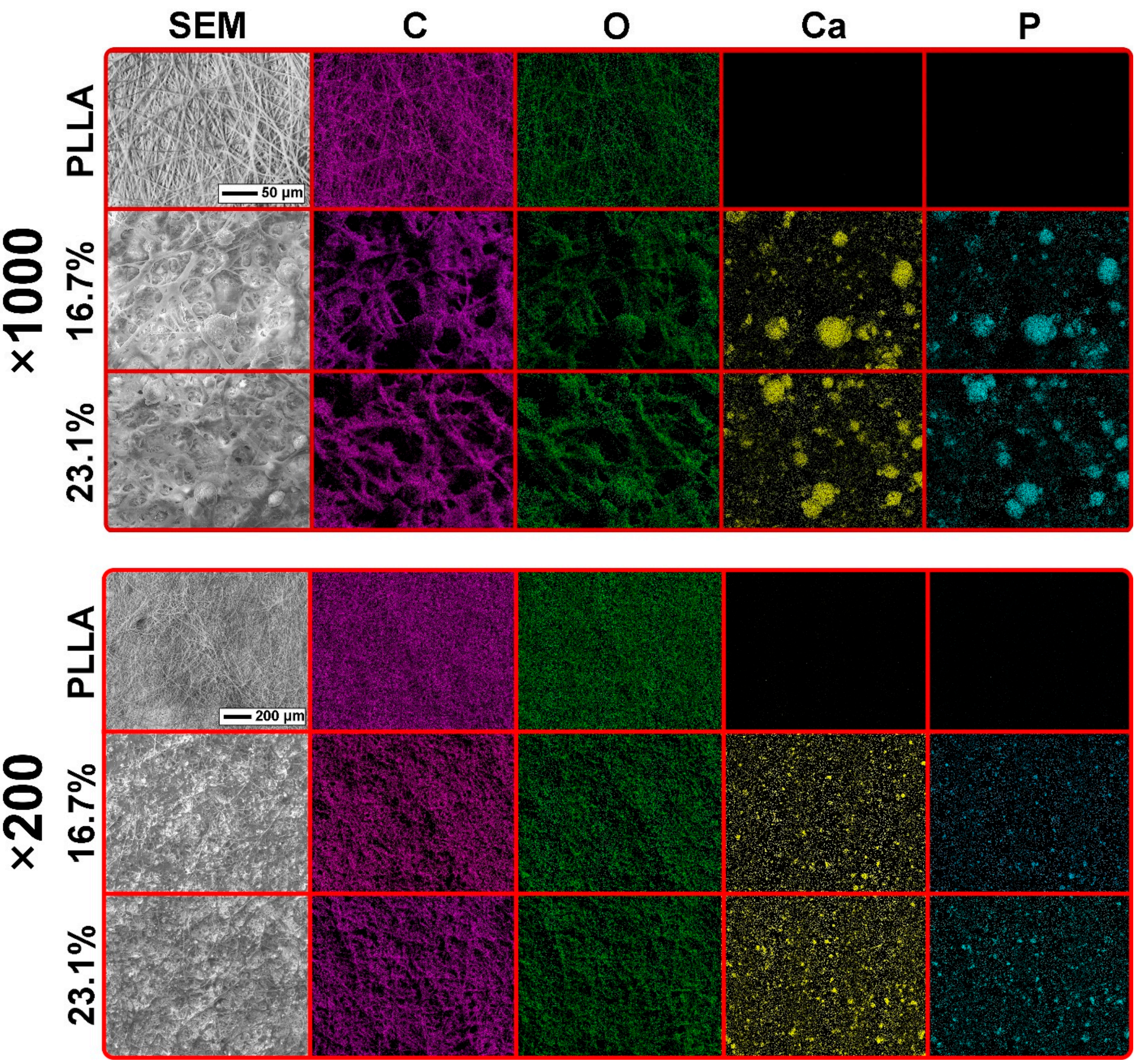

Figure S8. EDX mapping images (for the elements: C—carbon, O—oxygen, Ca—calcium, P—phosphorus) of the PLLA and PLLA/HAP scaffold samples examined, taken at ×1000 and ×200 magnifications. Note: "16.7%" refers to the electrospun PLLA/HAP scaffolds containing 20 wt.% HAP relative to the PLLA mass, and "23.1%" refers to the electrospun PLLA/HAP scaffolds containing 30 wt.% HAP relative to the PLLA mass.

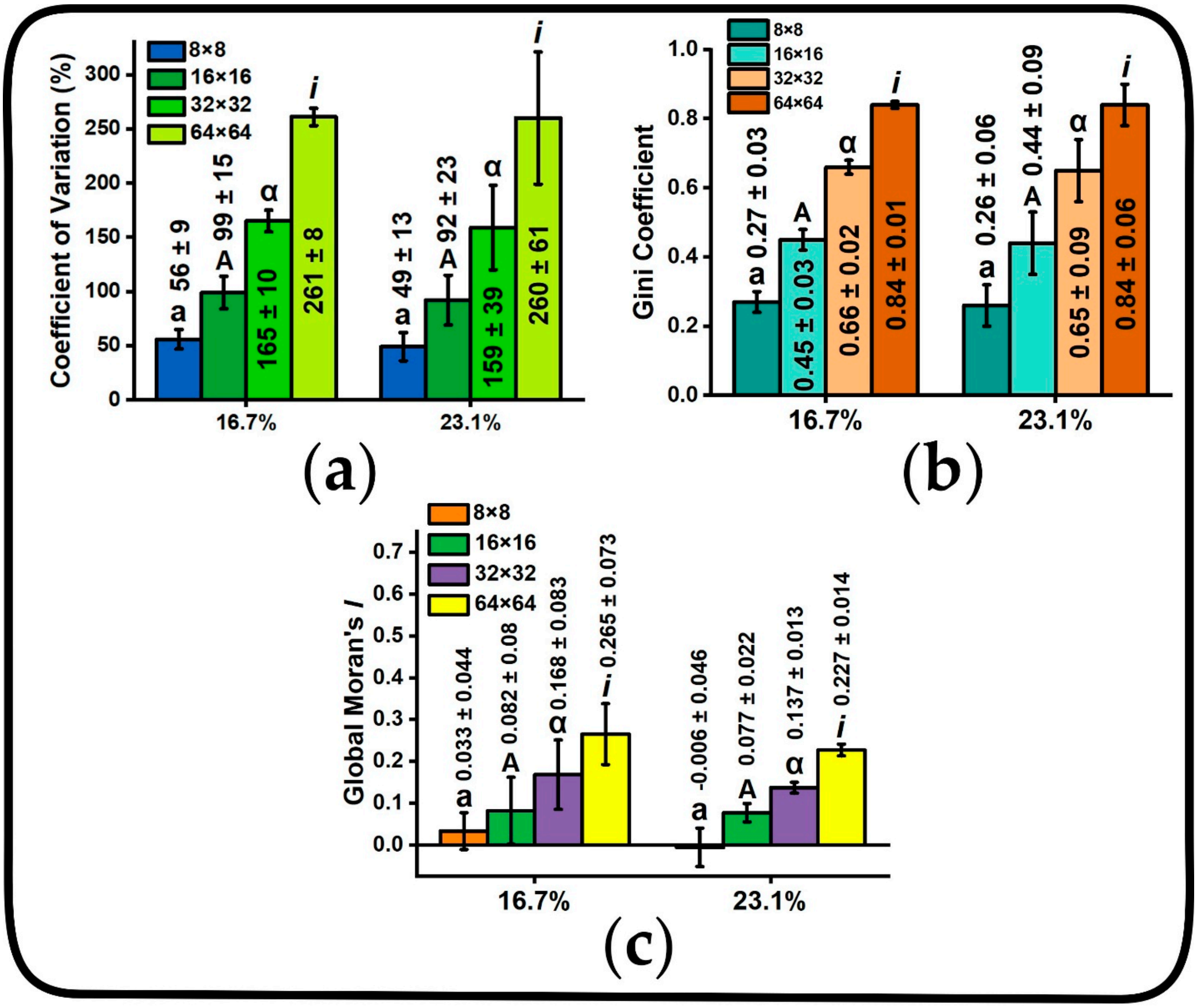

Figure S9. Quantitative assessment of hydroxyapatite particle distribution based on EDX mapping images of PLLA/HAP scaffolds acquired at ×200 magnification using different image partitioning schemes (grid sizes: 8×8, 16×16, 32×32, and 64×64): (a) coefficient of variation (%), (b) Gini coefficient, and (c) Global Moran's I. Note: The hydroxyapatite particle size distributions were obtained from BFM images acquired at ×10 magnification. "16.7%" denotes electrospun PLLA/HAP scaffolds containing 20 wt.% HAP relative to the PLLA mass, whereas "23.1%" denotes electrospun PLLA/HAP scaffolds containing 30 wt.% HAP relative to the PLLA mass.

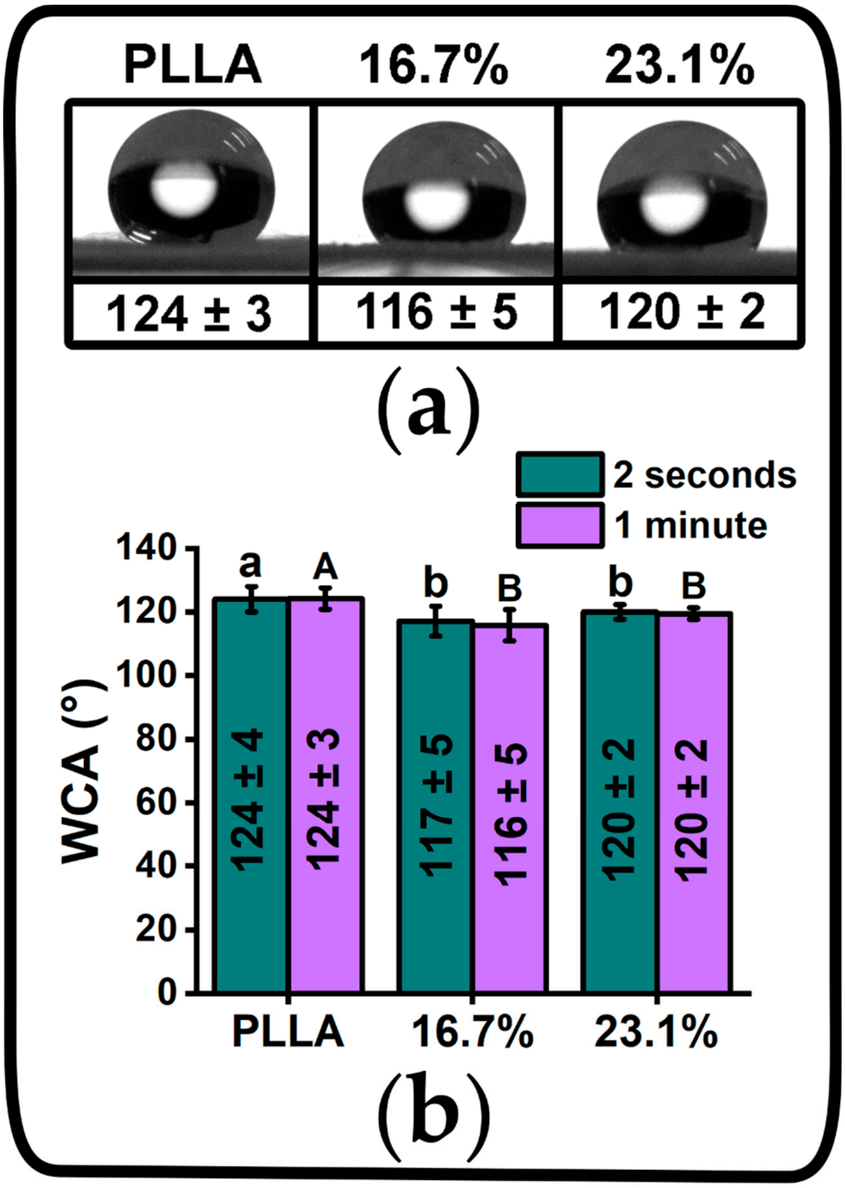

Figure S10. Wettability of PLLA and PLLA/HAP scaffolds: (a) digital photographs of water droplets after 1 min of interaction with the scaffold surface, (b) water contact angles after 2 s and 1 min of interaction of water droplets with surface of the scaffolds. Note: "16.7%" refers to the electrospun PLLA/HAP scaffolds containing 20 wt.% HAP relative to the PLLA mass, and "23.1%" refers to the electrospun PLLA/HAP scaffolds containing 30 wt.% HAP relative to the PLLA mass.

Table S1. Areal density and porosity of PLLA and PLLA/HAP scaffolds calculated via gravimetric method.

№	Sample	Areal density, mg/cm^2	Porosity values, %
1	PLLA	3.89 ± 0.73[a]	82.3 ± 0.5[a]
2	16.7%	8.86 ± 0.08[b]	74.9 ± 0.3[b]
3	23.1%	7.69 ± 0.21[c]	77.3 ± 0.8[c]

Note: Different lowercase letters within each column indicate statistically significant differences among samples ($p < 0.05$).

Table S2. Crystallite size and crystallinity degree for the prepared PLLA/HAP scaffold samples, which was estimated based on X-ray diffractograms.

№	Sample	Crystallite size of PLLA (200/110), nm	Crystallite size of HAP (211), nm	Crystallinity degree of PLLA, %
1	PLLA	-	-	-
2	16.7%	14.8 ± 0.5[a]	7.6 ± 0.4[a]	6.8 ± 0.4[a]
3	23.1%	14.5 ± 0.8[a]	6.7 ± 0.9[a]	3.7 ± 0.3[b]

Note: Different lowercase letters within each column indicate statistically significant differences among samples ($p < 0.05$).

Table S3. The relative area occupied by HAP particles on the surface of PLLA/HAP scaffolds, calculated using SEM and EDX mapping images.

№	Sample	Area occupied by HAP particles, %
1	PLLA	-
2	16.7%	12.3 ± 0.9[a]
3	23.1%	14.2 ± 1.2[b]

Note: Different lowercase letters indicate statistically significant differences among samples ($p < 0.05$).
